# When personality meets surprise: individual differences in memory for unexpected events

**DOI:** 10.3389/fpsyg.2025.1652428

**Published:** 2025-12-08

**Authors:** Alex Kafkas, Matt Westerman, Keith Cleto, Karolina Sabaityte, Katerina Sergi

**Affiliations:** Andrew Mayes Centre for Cognitive Neuroscience, School of Health Sciences, University of Manchester, Manchester, United Kingdom

**Keywords:** encoding, expectation, recollection, prediction errors, personality, anxiety, risk-taking, episodic memory

## Abstract

**Introduction:**

Expectation shapes memory formation, with unexpected stimuli typically eliciting richer encoding, greater associative processing, and superior recollection. Yet, the role of personality traits in modulating this effect is poorly understood, despite their potential influence on how individuals engage with and interpret environmental cues. This study investigated whether dispositional factors shape memory encoding for expected versus unexpected events.

**Methods:**

Across two experiments, participants learned symbol–stimulus contingencies that were later violated for a subset of stimuli at encoding, followed by a recognition memory test. Additionally, in Experiment 1 (*n* = 55), state/trait anxiety and risk-taking were measured, while in Experiment 2 (*n* = 142), participants completed the Big Five personality inventory.

**Results:**

In both experiments, unexpected events boosted subsequent recollection, but the magnitude of this benefit varied systematically with individual differences. Experiment 1 showed that individuals low in trait anxiety and risk-taking exhibited pronounced recollection benefits for unexpected events, an advantage that diminished in highly anxious or risk-taking individuals. Experiment 2 extended these findings, revealing that low extraversion and high negative emotionality attenuated the recollection advantage of unexpected events.

**Discussion:**

These findings demonstrate that personality traits dynamically shape how expectation guides memory formation and offer new insight into the interplay between dispositional factors and cognition. They also highlight important implications for both memory theory and applied contexts.

## 1 Introduction

The constant influx of novel information in everyday life highlights the brain’s fundamental need to update predictions and adjust to changing circumstances (Bar, 2009; Friston, 2010; Kafkas and Montaldi, 2015; Press and Yon, 2019). Such predictive processes not only guide perception (de Lange et al., 2018), but critically shape how new experiences are encoded into memory, creating updated representations for future reference (Frank and Kafkas, 2021). Although the influence of expectation on memory formation is well documented, an important yet largely neglected area of research concerns how individual differences, such as anxiety, risk-taking tendencies, and other personality traits, modulate the impact of expectation on memory encoding. As discussed below, expectation may not exert a uniform influence on everyone but could be modulated by dispositional factors linked to how people engage with the stimuli around them.

Unexpected stimuli elicit neural and cognitive responses aimed at updating internal models of the environment, which helps to guide future behavior (Friston, 2010). From an evolutionary standpoint, efficiently processing and remembering unexpected events is advantageous. Novel stimuli often indicate opportunities for reward or potential threats, such as a predator’s sudden appearance, necessitating memory to facilitate a swift response (De Loof et al., 2018; Jang et al., 2019; Miendlarzewska et al., 2016). This is further heightened in the case of unexpected or surprising outcomes. Indeed, encountering unexpected stimuli typically triggers richer and more elaborate encoding strategies, as evidenced by increased visual exploration, longer fixation durations, and greater pupil dilation responses (Kafkas, 2021; Kafkas and Montaldi, 2015). Consequently, unexpected events are often remembered with greater detail and contextual richness, as indicated by vivid recollective experience (Foster and Keane, 2019; Greve et al., 2019; Reichardt et al., 2020; Schmidt and Schmidt, 2017).

However, the relationship between expectation and memory is not entirely straightforward. Bollinger et al. (2010) found that expected stimuli, facilitated by predictive cues, can also enhance perceptual processing and subsequently improve memory performance. Furthermore, memory may favor schema-congruent (equivalent to expected) stimuli, despite that schema-incongruent (unexpected) stimuli typically draw more attention at encoding (Bein et al., 2015; Liu et al., 2018; Van Kesteren et al., 2018). These contrasting findings may suggest that both expected and unexpected information can benefit memory, albeit, possibly through different mechanisms and memory systems. They may also emphasize that individual differences influence how expected and unexpected events are encoded into memory.

Recent studies have reconciled the seemingly contradictory findings by distinguishing different encoding pathways driven by expectation (Greve et al., 2019; Kafkas, 2021; Kafkas and Montaldi, 2018a). For example, violations of what is anticipated can bolster the retrieval of detailed contextual information (i.e., recollection), whereas events that align with expectations tend to rely more on gist-like familiarity memory (Kafkas and Montaldi, 2018a; for the distinction between memory types that support recognition memory see Montaldi and Kafkas, 2024; Yonelinas et al., 2010). Neuroimaging research supports this distinction by showing that unexpected stimuli reliably engage hippocampal computations, partly via increased connectivity with midbrain regions such as the ventral tegmental area (Frank et al., 2020; Kafkas and Montaldi, 2015; Wittmann et al., 2005). Dopaminergic signaling from the midbrain could modulate hippocampal plasticity when unexpected events are encountered (Clos et al., 2019). This leads to richer associative encoding and larger pupil dilation responses – a marker of heightened cognitive engagement (Van Der Wel and Van Steenbergen, 2018) and memory encoding (Kafkas, 2021). Therefore, this evidence supports that both expected and unexpected events can enhance memory, albeit through distinct mechanisms. Expected stimuli are processed more efficiently due to attention-mediated perceptual facilitation, which increases the sense of similarity between current inputs and stored representations and thus selectively enhances familiarity-based recognition. In contrast, unexpected stimuli elicit prediction errors that trigger hippocampal pattern separation and deeper encoding, supporting recollection-based retrieval (Kafkas and Montaldi, 2018a, 2018b; Kafkas, 2021).

While predictive-processing accounts provide a useful framework for understanding how expectation violations shape encoding, most studies have treated participants as cognitively uniform, focusing on average effects of surprise or prediction error. However, some evidence suggests that dispositional traits modulate attentional breadth, uncertainty tolerance, and motivational engagement with novelty (Blais and Weber, 2006; Robinson, 2004; Smillie et al., 2019; Zsido et al., 2020). This indicates that individual differences may determine whether expectancy violations enhance or impair memory, a question that remains underexplored in existing models of predictive learning and episodic encoding. Therefore, encoding responses to unexpected or expected stimuli may not be uniform across individuals but instead depend on their traits and cognitive styles. Although direct evidence on how personality traits modulate expectation-based memory is scarce, several theoretical frameworks suggest plausible mechanisms. Predictive-processing accounts propose that individuals differ in the precision weighting they assign to prediction errors, influencing how surprising information is encoded (Friston, 2010; Kafkas and Montaldi, 2018b). In parallel, personality models such as Trait Activation Theory (Tett and Burnett, 2003) and the Cognitive–Affective Personality System (Mischel and Shoda, 1995) predict that dispositional tendencies become manifest in contexts that activate trait-relevant motivational and emotional processes. From this perspective, traits related to anxiety, risk-taking, and extraversion may modulate how individuals respond to expectancy violations during learning, thereby shaping memory outcomes.

For instance, heightened anxiety can narrow attentional focus, bias attention toward perceived threats, and disrupt cognitive control, potentially undermining detailed memory encoding (Barkasi and Rosen, 2020; Zsido et al., 2020). Conversely, individuals with high risk-taking tendencies might preferentially engage with unexpected stimuli, viewing them as rewarding or exciting opportunities, thereby promoting exploratory behaviors and detailed encoding strategies (Blais and Weber, 2006; Figner and Weber, 2011; Huo et al., 2020). Moreover, personality traits such as extraversion and openness to experience may further influence how individuals process novel and unexpected information. Highly extroverted individuals often seek novel stimuli (Ausmees et al., 2022; Stolz et al., 2023), potentially facilitating engagement with unexpected events which may lead to enhanced recollection. In contrast, negative emotionality or high trait anxiety might restrict attention and limit detailed processing of unexpected information (Klaming et al., 2017), consequently diminishing the memory advantage typically associated with expectation violations.

To address this critical gap in the literature, we conducted two experiments investigating how various personality factors modulate expectation-based memory encoding. Participants first learned predictive symbol–stimulus associations. These were designed to allow deriving expected (rule-consistent) or unexpected (rule-inconsistent) outcomes at encoding. A separate set of stimuli were used in the encoding task, some aligning with previously learned expectations and others violating them. Participants’ memory for the encoded stimuli was tested after a short delay using a recognition memory test. Experiment 1 focused specifically on anxiety and risk-taking tendencies, while Experiment 2 extended this approach to include the broader Big Five personality dimensions (Eysenck, 1981; Soto and John, 2017). We first examined state/trait anxiety and risk-taking because both directly capture tolerance to uncertainty and behavioral responses to prediction error — mechanisms central to expectation-driven encoding. In Experiment 2, we extended this approach to a broader personality framework by assessing the Big Five dimensions, allowing us to test whether the influence of dispositional factors on expectation-based memory generalizes beyond specific affective and risk-related traits.

Overall, we hypothesized that high levels of anxiety and negative emotionality would attenuate the recollection boost typically observed for unexpected stimuli, whereas increased risk-taking tendencies and extraversion would enhance it. This hypothesis follows from evidence that anxious or negatively emotional individuals exhibit narrowed attention and avoidance of uncertainty (Zsido et al., 2020), whereas risk-takers and extraverts typically show heightened engagement with novel and surprising stimuli (Blais and Weber, 2006; Smillie et al., 2019). Given the scarcity of prior evidence, the effects of other personality traits were explored without *a priori* directional hypotheses. Overall, this exploration aimed to uncover insights into the flexible interplay between expectation-driven memory processes and individual dispositional factors.

## 2 Materials and methods

### 2.1 Participants

In both experiments, ethical approval was obtained from the University of Manchester research ethics committee. Participants provided informed consent prior to completing the experiments. Their details remained confidential, and their anonymity and right to withdraw was ensured, in compliance with ethical research conduct. Participants had normal vision and no present experience or history of psychiatric or neurological disorders. In Experiment 1, 60 undergraduate students from the University of Manchester were recruited, in exchange for course credits. Five participants were excluded from all data analyses due to not completing the experiment (1 participant) and insufficient learning of the rules in the rule learning task, as indicated by poor accuracy (4 participants with accuracy less than 60%; this cut-off was determined prior to data collection and is consistent with other published studies with similar methodology, e.g., Kafkas and Montaldi, 2018a). One participant identified expectation to be a key manipulation but was retained in the final analysis. The final sample thus comprised of 55 participants (7 Male, 48 Female), with a mean age of 19.23 years (SD = 1.30). An *a priori* power analysis indicated that 50 participants were required to replicate the expectation effect on memory (Kafkas and Montaldi, 2018a), while incorporating the between-subject factors of anxiety and risk-taking (effect size *f^2^* = 0.71, power = 0.90).

In Experiment 2, 154 young adults were recruited based on an a priori power calculation for a regression analysis with five predictors, based on the results from Experiment 1. Four participants were excluded due to insufficient learning of the rules in the rule-learning task (less than 60% accuracy), and an additional eight participants were excluded as outliers performing below chance in the recognition memory task. The final sample consisted of 142 participants (27 Male, 114 female, while 1 participant self-identified as ‘other’), with a mean age of 19.23 years, (SD = 1.16), who participated in the study for course credits.

### 2.2 Materials, design and stimuli

The stimuli included 244 greyscale images of man-made and natural objects (500 × 375 pixels), along with six line-drawn symbols, which were used for the rule learning task and as contextual cues in the encoding task, following previously published work (Kafkas, 2021; Kafkas and Montaldi, 2018a). The experiment was programmed and run on Gorilla^1^, while the experimenters communicated with the participants remotely via Zoom (zoom.us; only for Experiment 1). Experiment 1 employed a mixed factorial design with expectation (expected, unexpected) and memory type (familiarity, recollection) as within-subjects factors, and individual difference measures (state and trait anxiety, and risk-taking behavior) each examined separately as between-subjects factors (high vs. low, based on a median split). Experiment 2 used a similar mixed design, with expectation and memory type as within-subjects factors, and personality as a between-subjects factor. Each of the five Big Five personality dimensions (openness, conscientiousness, extraversion, agreeableness, and neuroticism) was analyzed separately by grouping participants into high and low trait levels using a median split (see Data analyses).

To obtain measures of anxiety and risk taking, a modified version of Spielberger’s (1983) State–Trait Anxiety Inventory (STAI), the STAI-5 (Zsido et al., 2020), and The Domain Specific Risk-taking Scale (DOSPERT) (Blais and Weber, 2006) were used. The STAI-5 consists of a 4-point Likert scale for both state anxiety (the STAIS-5) and trait anxiety (the STAIT-5), with 5 items, each. The STAIS-5 required participants to rate how they felt at the time, responding to statements like ‘I feel frightened’. Conversely, the STAIT-5 asked participants to rate how they generally felt, with items such as ‘I worry too much over something that does not really matter’. For each item on both STAI scales, participants chose from four options ranging from ‘Not at all’ (1) to ‘Very much so’ (4). The scales were scored by summing participants’ ratings across all five items. Both scales have shown excellent internal consistency (Cronbach’s alpha = 0.90 and 0.82, respectively) and strong correlations with full STAI (*r* = 0.95; Zsido et al., 2020). The DOSPERT scale comprises 30 items, requiring participants to rate the likelihood of engaging in behaviors such as ‘Taking a skydiving class’ on a 7-point Likert scale (1 = ‘Extremely unlikely’, 7 = ‘Extremely likely’). The scale has a high overall internal consistency (Cronbach’s alpha = 0.85; Blais and Weber, 2006) and temporal stability (test–retest *r* = 0.58–0.87). Construct validity is supported by its five-domain factor structure and by positive correlations with sensation seeking and impulsivity, and negative correlations with risk aversion and harm avoidance (Weber et al., 2002; Weller et al., 2018). Participants’ scores in this scale were calculated by summing their ratings across all 30 items.

Experiment 2 was completed fully online (without experimenters on video call) and after the experimental task, the Big Five Inventory-2 (BFI-2; Soto and John, 2017) was administered to the participants to derive measures across the 5 personality traits. BFI-2 is a revised version of the original Big Five Inventory, contains 60 questions, which are designed to measure the five major domains of personality: extraversion, agreeableness, conscientiousness, negative emotionality (formerly neuroticism), and open-mindedness (formerly openness to experience). It shows excellent internal consistency (*α* = 0.84–0.88 at the domain level), good temporal stability (test–retest *r* ≈ 0.80 over 6–8 weeks), and strong structural, convergent, and criterion validity (Soto and John, 2017). The BFI-2 is scored by averaging responses to the 60 items, which are grouped into the five broad personality domains. Each domain is assessed using 12 items, with participants rating each item on a 5-point Likert scale (1 = Disagree strongly; 2 = Disagree a little; 3 = Neutral; 4 = Agree a little; 5 = Agree strongly). The final scores for each domain reflect the participant’s standing on that personality dimension, with higher scores indicating a stronger presence of the trait.

### 2.3 Procedure

In Experiment 1, upon joining a Zoom call with the experimenter, participants received a link and unique participant ID to access and complete the experiment on Gorilla. The experimenter remained on the Zoom call throughout the session. Participants completed a rule learning task, an encoding task, and a recognition memory task, in that order. At the end of the session, they completed the STAIS-5, STAIT-5, and DOSPERT scales, followed by a debriefing. In Experiment 2, participants completed the study fully online and after the experimental tasks (rule learning, encoding and memory tasks), the BFI-2 was completed. As participants in Experiment 1 rarely required clarification of the written instructions, Experiment 2 was conducted entirely online. The experiment also included four attention-check trials—one in each main block (rule learning in cycle 3, encoding, filler, and recognition)—requiring an instructed button press to verify participants’ engagement throughout the study. Participants who failed more than two checks were excluded and replaced to maintain the target sample size. This occurred rarely and only one participant was replaced, while over 80% of participants passed all four attention checks.

#### 2.3.1 Rule learning task

**Figure 1:**
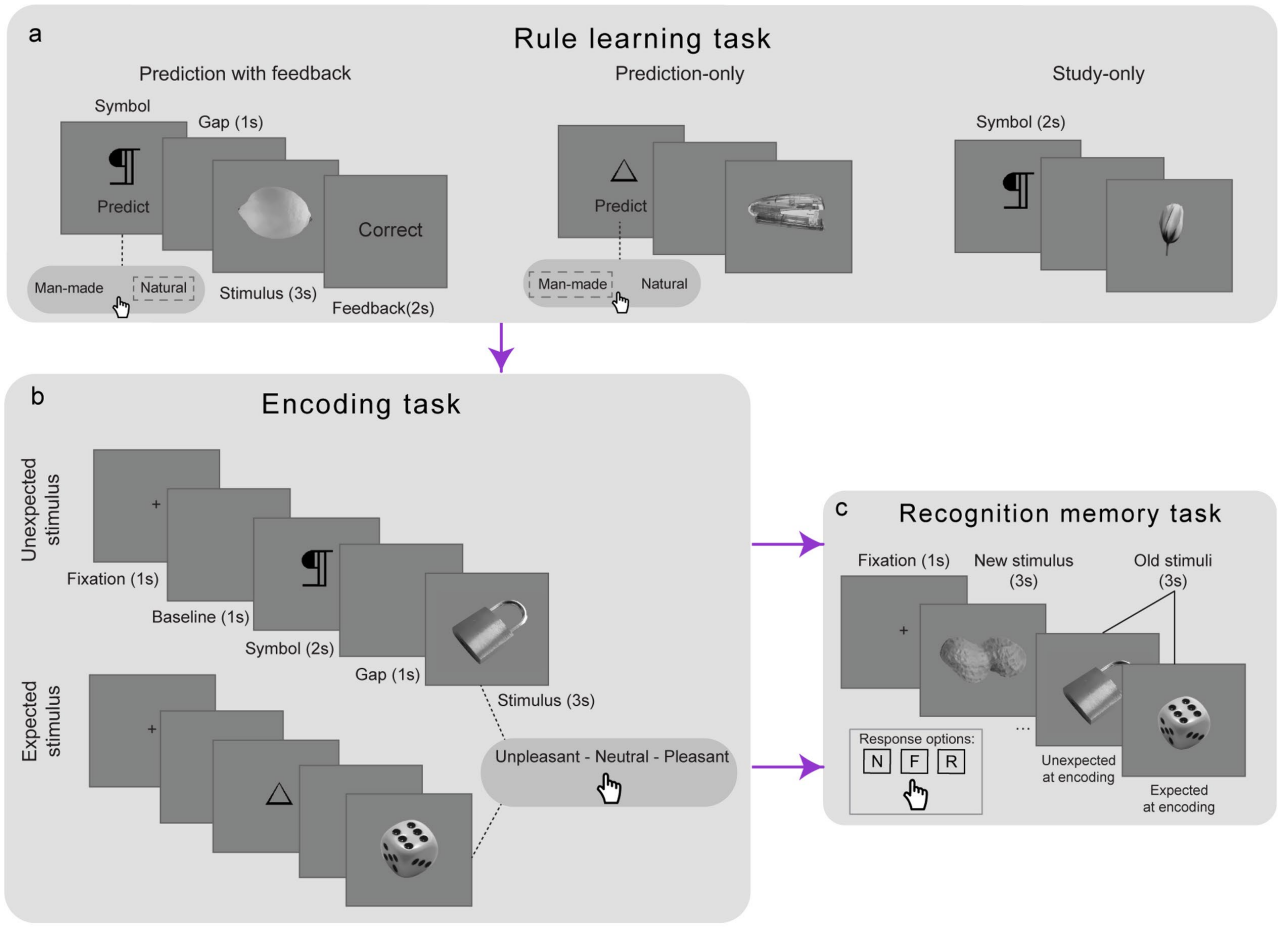
Design of the experiments. A rule-learning task **(a)** was used to establish expectations regarding the symbol–stimulus sequence in each trial across three cycles (prediction with feedback, prediction-only and study-only). These expectations were then manipulated in the encoding task **(b)** for a new set of stimuli, with some stimuli violating the previously established rules (unexpected) and others upholding them (expected). Memory for the encoded stimuli was subsequently tested in a recognition memory task **(c)** and participants were asked to identify each stimulus as new (N), familiar (F) or recollected (R).

To ensure that expectations could be reliably manipulated at encoding, participants first learned symbol-stimulus (SS) associations in a rule learning task (Figure 1), as established in previously published work (Kafkas, 2021; Kafkas and Montaldi, 2018a). Six abstract symbols were used: three predicted man-made items and three predicted natural items. The mapping between symbols and categories was counterbalanced across participants. The task comprised 72 trials, divided into three successive cycles. In Cycle 1 (prediction with feedback) 36 trials were presented. In each trial participants saw a symbol and prompted to predict whether a ‘man-made’ or ‘natural’ item would follow. After selecting an option, the corresponding stimulus was presented for 3 s. A feedback screen, shown for 2 s, indicated whether participant’s prediction was correct or incorrect. In Cycle 2 (prediction-only)18 trials were presented. The same prediction procedure was followed as in Cycle 1, but feedback was omitted. Finally, in Cycle 3 (study-only) another 18 trials were presented and participants viewed each SS pair without making a prediction. Instead, they were instructed to study each SS sequence. In this block each symbol appeared for 2 s, followed by a one-second gap, and then the stimulus for 3 s. The final cycle was designed to facilitate a smooth transition to the subsequent encoding task, which employed a comparable trial sequence comprising the same key elements. This design facilitated the formation of robust expectations regarding the event sequence within each trial, enabling effective manipulation of these expectations in the subsequent encoding task.

#### 2.3.2 Encoding and expectation manipulation

Participants proceeded to the encoding task (Figure 1), where the same symbols from the rule-learning task were paired with a new set of stimuli. In this phase, participants rated the pleasantness of each stimulus, a semantic decision designed to enhance encoding by directing attention to the stimuli. The six symbols were each paired with 20 stimuli, resulting in 120 randomized trials in this task. Each trial began with a one-second fixation cross, followed by a symbol (2 s), a gap (1 s), and then the stimulus (3 s), during which participants rated it as unpleasant, neutral, or pleasant by pressing one of three designated arrow keys. Crucially, the expectation status was manipulated: 40% (48) of the stimuli violated the learned rule and were therefore unexpected (e.g., a symbol typically cueing a natural item preceded a man-made item, and vice versa), while the remaining 60% (72) conformed to the rule (expected stimuli). Following this, participants completed a numerical distractor task, evaluating 34 equations presented for 10 s each as correct or incorrect.

#### 2.3.3 Recognition memory task

Prior to starting the recognition memory task (Figure 1), participants in Experiment 1 were instructed to unmute their Zoom call, during which the experimenters provided verbal explanations of the definitions of the response options (new, familiar and recollected). In contrast, participants in Experiment 2 received the recognition memory instructions on-screen and were then required to write their own definitions of the response options in a free-text field, as they applied to the task. All participants in the final sample demonstrated a good understanding of when to use the responses based on their memory experience. Generally, participants were trained to identify a stimulus as familiar if they recognized it without retrieving specific details, or as recollected if they could recall associative details (e.g., thoughts or incidental details such as its order among the stimuli) (Montaldi and Kafkas, 2024). A practice block of five trials, using stimuli from the rule-learning task and some novel items, was provided to reinforce these definitions and ensure participants could distinguish between the different memory experiences. In the main recognition memory task, 170 randomized trials were presented: 120 target stimuli from the encoding task and 50 foils (new stimuli not previously encountered). Each trial began with a fixation cross (1 s), followed by the stimulus and a prompt for participants to categorize it as new, familiar, or recollected within a 3-s window using three arrow keys as response buttons.

#### 2.3.4 Questionnaires

Lastly, participants completed the STAIS-5, STAIT-5, and DOSPERT scales in this order (Experiment 1) or the BFI-2 (Experiment 2). After completing the questionnaires, participants were debriefed and asked to describe the experiment’s aim and whether they noticed the expectation manipulation during the encoding task, specifically through violations of the SS associations they had learned. In Experiment 1, one participant correctly identified the rule violations as the study’s key manipulation but was still included in the final analyses. All participants were then informed of the experiment’s aims and received course credits for their participation.

### 2.4 Data analyses

In both experiments, two one-way repeated-measures ANOVAs were performed to examine participants’ prediction accuracy (% correct) and response times (in milliseconds) across nine symbol repetitions in cycles 1 and 2 of the rule-learning task. In addition, a two-way repeated-measures ANOVA, with pleasantness rating (unpleasant, neutral, pleasant) and expectation status (expected, unexpected) as factors, was conducted on the proportion of trials participants rated as unpleasant, neutral, or pleasant in the encoding task. Recognition memory responses were categorized using signal detection outcomes (familiarity hits, recollection hits, familiarity false alarms, recollection false alarms, correct rejections, and misses). Memory performance for familiarity and recollection was computed by subtracting the relevant false alarm rate from the hit rate. Sensitivity (d’) was also computed as z(hit rate) – z(FA rate), but as it yielded comparable patterns across analyses, these results are not reported separately. Another two-way repeated-measures ANOVA tested the effects of expectation status (expected, unexpected) and memory type (familiarity, recollection) on memory performance (Hits-FA). In Experiment 1, three additional three-way mixed ANOVAs were run on memory performance. Expectation status (expected, unexpected) and memory type (familiarity, recollection) served as within-subjects factors, while state anxiety, trait anxiety, and risk-taking were each included as a between-subjects factor (high vs. low). Participants were split using median values: 33 scored high on state anxiety (≥6) and 22 low (range 5–12); 25 scored high on trait anxiety (≥10) and 30 low (range 6–19); 27 scored high on risk-taking (≥96) and 28 low (range 64–145).

To further examine the effects and enhance statistical power by leveraging the relatively large number of trials (despite the smaller sample size in Experiment 1), we fitted a Rescorla-Wagner reinforcement learning model (Yau and McNally, 2023) to trial-by-trial prediction data (from the rule-learning task), using a single learning rate (*α*) for each participant which represents the extent to which new information changes participants predictions. The single learning rate (*α*) ranges from 0 to 1, with lower values indicating minimal updating (i.e., reliance on prior beliefs) and higher values reflecting rapid adjustment based on new outcomes.

In this analysis, we estimated prediction error (PE) on a trial-by-trial basis using participants’ rule-learning accuracy. To model participants’ evolving expectations during the rule-learning phase, we fitted a Rescorla-Wagner (RW) reinforcement learning model to each participant’s sequence of prediction responses using Python (version 3.13) with Pandas (version 2.2) and NumPy (version 2.3). On each trial, participants predicted an object category based on a symbolic cue and received feedback. These binary outcomes were used to update the model’s estimate of associative strength (*V*) between each symbol and its associated category (see Equation 1). Specifically, for each participant, the learning rate parameter *α* was estimated using a grid-search procedure. The learning rate parameter *α* was constrained to the interval [0, 1] and evaluated in 0.01 increments (101 candidate values). For each α, associative strengths *V* were initialized at 0.5 on the first occurrence of each symbol and updated sequentially across trials (sorted by trial index) according to the standard Rescorla–Wagner rule:


(1) $$V_{t+1}=V_{t}+a(R_{t}-V_{t})$$

where *R_t_* is the observed outcome (1 for correct, 0 for incorrect), and *α* is a learning rate parameter controlling the speed of updating. Model fit was quantified using the sum of squared prediction errors (SSE) across trials, and the α that minimized SSE was selected as the best-fitting learning rate for that participant. This approach imposes fixed parameter bounds, no priors, and requires no convergence settings or stopping criteria. The fitted *α* was then used to compute trial-by-trial PE values (see Equation 2), defined as:


(2) $$PE_{t}=R_{t}-V_{t}$$

Trial-wise PE values and final α estimates were merged back into the behavioral data (subsequent memory outcomes) for further regression analyses. This allowed us to assess how prior learning (in the rule-learning) and expectation violations (at encoding) influence later memory performance. The full code used for grid search, parameter estimation, and extraction of prediction-error time series is available at the OSF repository associated with this study (see Availability of data and materials).

PE distributions were broad and symmetrical, with no extreme outliers, and qualitative inspection of prediction trajectories confirmed alignment with rule learning accuracy trends (see Supplementary Figure 1). These PE estimates were then aligned with the corresponding items during the encoding phase and merged with subsequent recognition memory data. To assess how trial-by-trial learning influenced later memory, we used logistic regression models in which the dependent variable was binary memory accuracy. For this analysis, we collapsed Familiar (F) and New (N) responses into a single “non-recollected” category, contrasting them with Recollected (R) responses. This binary outcome allowed us to isolate recollection as the memory outcome of interest while also ensuring stable model estimation and sufficient statistical power for testing interactions. Therefore, we assessed whether each item was correctly identified as old (hit) or incorrectly identified as new (miss). Separate models also assessed binary recollection outcomes (recollected vs. not recollected). These models included PE values, expectation status (expected vs. unexpected), and their interaction as predictors. This approach enabled us to determine whether higher prediction error during learning increased the likelihood of correct recognition, particularly for unexpected stimuli. To evaluate model diagnostics, we calculated McFadden’s pseudo-R^2^ and variance inflation factors (VIFs), which indicated acceptable fit and no evidence of multicollinearity. Additional regressions tested trait anxiety and risk-taking as moderators.

In Experiment 2, each of the Big Five personality dimensions (extraversion, agreeableness, conscientiousness, negative emotionality, open-mindedness) was used as a between-subjects factor in five separate three-way ANOVAs on memory performance (Hits-FA), again with expectation status (expected, unexpected) and memory type (familiarity, recollection) as within-subjects factors. Participants were classified into high or low categories based on median splits of each trait’s score range. For extraversion (score ranges: 1.60–4.90), 69 participants scored high (≥3.25), and 73 scored low. For agreeableness (score ranges: 2.00–4.70), 71 participants scored high (≥3.60), and 71 scored low. For conscientiousness (score ranges: 1.40–5.00), 70 participants scored high (≥ 3.30), and 72 scored low. For negative emotionality (score ranges: 1.20–4.90), 69 participants scored high (≥ 3.30), and 73 scored low. Finally, for open-mindedness (score ranges: 1.80–5.00), 72 participants scored high (≥ 3.80), and 70 scored low. Multiple regression analyses were also conducted, as the sample size was sufficiently larger in Experiment 2, using the continuous personality scores as factors predicting memory performance (Hits-FA), separately for expected and unexpected stimuli (i.e., expected familiar, expected recollected, unexpected familiar, unexpected recollected). Regression coefficients (B) representing unstandardized estimates of the relationship between each predictor and the dependent variable are reported in the Results as part of this analysis. Collinearity among the five predictors was very low as indicated by the Variance Inflation Factor (all VIFs < 1.16).

Prior to conducting the analyses, data distributions were examined for normality and homogeneity of variance. Normality was confirmed using the Shapiro–Wilk test, which revealed no significant deviations from normality across the dependent variables. Equality of variance was assessed with Levene’s test for all between-subjects factors, with no violations detected. Significant results across all the ANOVAs were further explored using Bonferroni-corrected pairwise comparisons or simple main effects (where appropriate), while Greenhouse–Geisser corrections were applied in case of sphericity violations (*p*-values noted as *p_GG_*). Response times were analyzed using the same variables and procedures as described above. Effect sizes were reported for all analyses, using partial eta squared (*η*_p_^2^) for ANOVAs and Cohen’s *d* for *t*-tests. The significance level for all analyses was set at *p* < 0.05.

## 3 Results

### 3.1 Rule learning task

**Figure 2:**
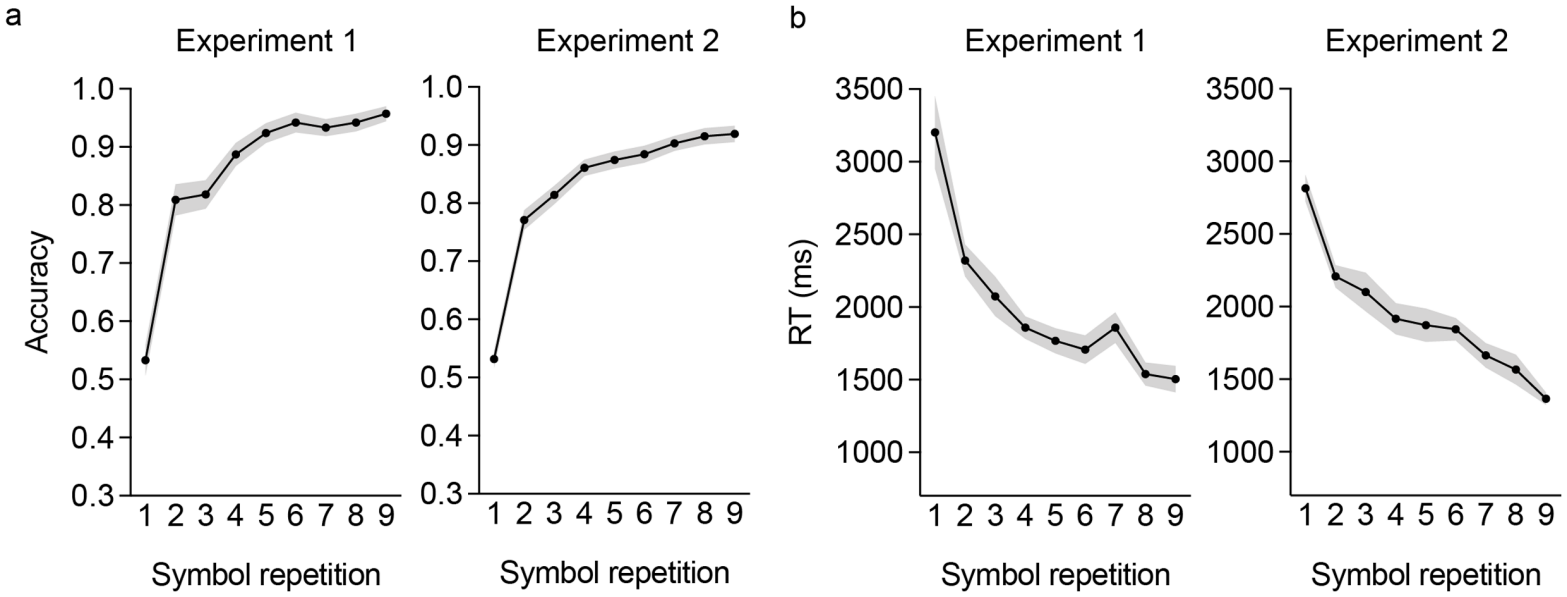
Prediction accuracy and response time across symbol repetition in the rule-learning task in the two experiments. Prediction accuracy increased with repeated presentations of the symbols **(a)**, while response times decreased across the repetition of the symbols **(b)**. Shaded areas in the plots show the standard error of the mean.

Participants demonstrated above-chance levels of performance in the rule learning task (one-sample *t*-tests against chance accuracy of 50%; Exp 1: *t*(54) = 29.37, *p* < 0.001; Exp 2: *t*(141) = 33.56, *p* < 0.001, *d =* 2.82), with an average accuracy of 86.09% (SD = 9.11; Exp 1) and 84.01% (SD = 12.07; Exp 2). Mean prediction accuracy (i.e., percent of correct prediction in cycles 1 and 2 of the task) and response times (ms) across repetitions of each symbol (9 repetitions; NB: the additional 3 presentations of each symbol did not require a prediction) from both experiments, are depicted in Figure 2. Accuracy differed significantly across symbol repetitions, [one-way ANOVA on prediction accuracy with symbol repetition as withing-subjects factor; Exp 1: *F*(8,432) = 63.94, *p* < 0.001, *η*_p_^2^ = 0.54; Exp 2: *F*(8,1,128) = 123.26, *p* < 0.001, *η*_p_^2^ = 0.47] characterized by a significant linear increase (polynomial contrast) in accuracy with each symbol repetition, [Exp 1: *t*(432) = 18.30, *p* < 0.001, *d* = 1.76; Exp 2: *t*(1128) = 26.13, *p* < 0.001, *d* = 1.56]. Similarly, prediction RTs across symbol repetitions also differed significantly [Exp 1: *F*(8,440) = 23.53, *p* < 0.001, *η*_p_^2^ = 0.30; Exp 2: *F*(8,1,128) = 39.08, *p* < 0.001, *η*_p_^2^ = 0.22] with a significant linear reduction in RTs with each symbol repetition [Exp 1: *t*(440) = −11.63, *p* < 0.001; Exp 2: *t*(1128) = −15.25, *p* < 0.001]. Collectively these findings indicate effective generation of expectations for correct SS sequences in the rule learning task of both experiments.

### 3.2 Encoding task

The mean proportions of pleasantness ratings (unpleasant, neutral, or pleasant) at encoding for expected and unexpected stimuli across the two experiments are presented in Supplementary Table 1 and were analyzed using a repeated-measures ANOVA with pleasantness and expectation as within-subjects factors. No significant difference was found in rating proportions between expected and unexpected stimuli [main effect of expectation; Exp 1: *F*(1,54) = 2.32, *p* = 0.13, *η*_p_^2^ = 0.04; Exp 2: *F*(1,140) = 1.12, *p* = 0.29, *η*_p_^2^ = 0.01]. Across pleasantness ratings, there was no significant difference in the proportions of unpleasant, neutral, and pleasant responses in Experiment 1 [*F*(2,52) = 3.52, *p* = 0.08, *η*_p_^2^ = 0.04], whereas the same comparison was significant in Experiment 2 [*F*(2,280) = 35.38, *p* < 0.001, *η*_p_^2^ = 0.20], indicating a lower proportion of unpleasant responses relative to neutral and pleasant responses (all *p*s < 0.001). The interaction between pleasantness rating and expectation status was not significant [Exp 1: *F*(2,108) = 1.52, *p* = 0.22, *η*_p_^2^ = 0.03; Exp 2: *F*(2,280) < 1], indicating that pleasantness ratings at encoding were not affected by the expectation status of the stimuli.

### 3.3 Effect of expectation on subsequent memory

**Figure 3:**
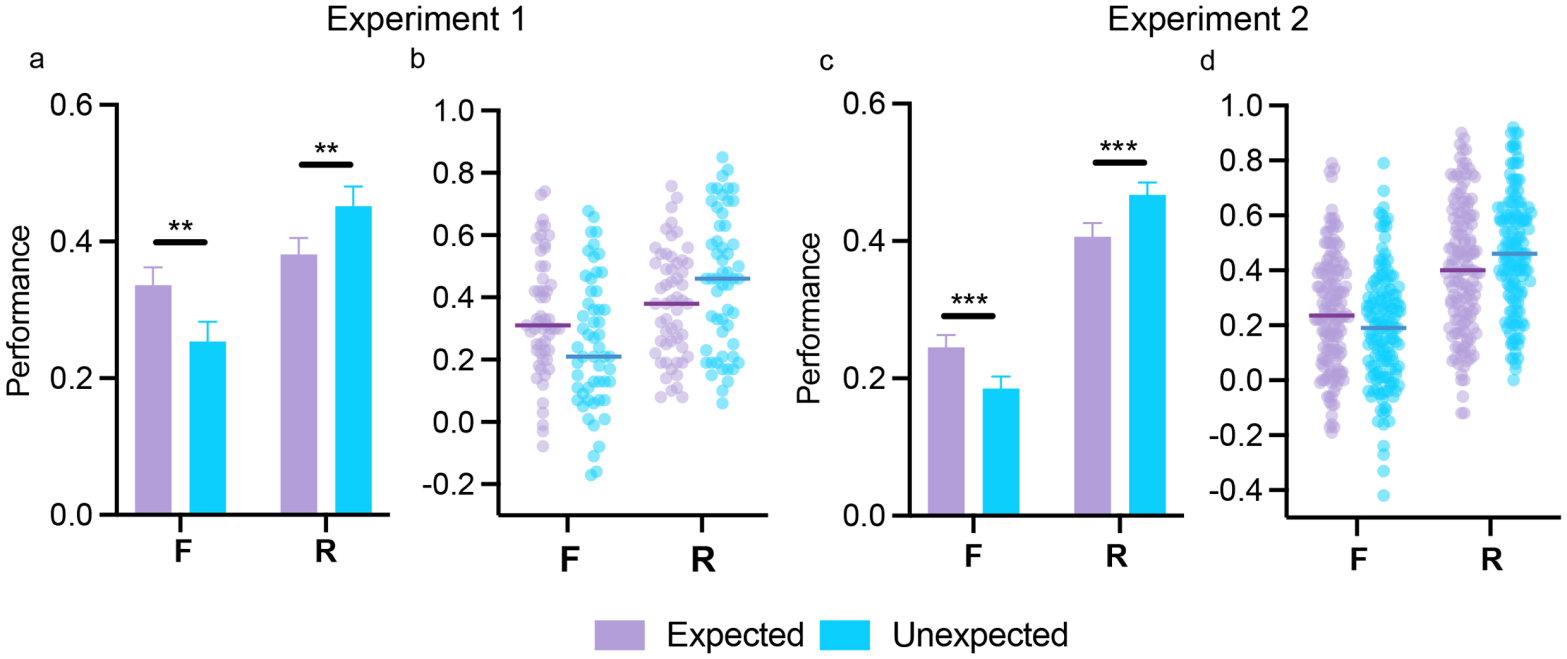
The effect of expectation on subsequent memory in Experiments 1 and 2. The overall effect of expectation on subsequent memory performance, separated by reported memory type (F/R), is shown in **(a,c)**, for Experiments 1 and 2, respectively. Individual scatterplots of memory performance depending on memory type (F/R) for expected and unexpected stimuli are presented in **(b,c)** for Experiments 1 and 2, respectively. In **(b,c)**, each point represents a participant’s score, while group means are indicated by the horizontal bars. Error bars in **(a,c)** indicate the standard error of the mean. **p < 0.01; ***p < 0.001.

Supplementary Table 2 presents the proportion of hits, false alarms, misses, and correct rejections for expected and unexpected stimuli in both experiments. The repeated-measures ANOVA (with expectation and memory type as within-subjects factors) on memory performance (hit rates – false alarm rates) showed similar performance between expected and unexpected stimuli [main effect of expectation; Exp 1: *F*(1,54) = 2.12, *p* = 0.15, *η*_p_^2^ = 0.04; Exp 2: *F*(1,141) = 0.05, *p* = 0.82, *η*_p_^2^ = 0.01]. Recollection responses were associated with significantly higher performance than familiarity responses [main effect of memory type; Exp 1: *F*(1,54) = 6.54, *p* = 0.013, *η*_p_^2^ = 0.11; Exp 2: *F*(1,141) = 54.66, *p* < 0.001, *η*_p_^2^ = 0.28]. Notably, differences in memory performance between familiarity and recollection responses varied by expectation status [expectation by memory type interaction; Exp 1: *F*(1,54) = 16.59, *p* < 0.001, *η*_p_^2^ = 0.24; Exp 2: *F*(1,141) = 22.14, *p* < 0.001, *η*_p_^2^ = 0.14]. As shown in Figure 3, *post-hoc* tests revealed that familiarity performance was higher for expected relative to unexpected stimuli [Exp 1: *t*(54) = 4.30, *p* < 0.001, *d* = 0.41; Exp 2: *t*(141) = 4.55, *p* < 0.001, *d* = 0.27], while recollection performance was higher for unexpected relative to expected stimuli [Exp 1: *t*(54) = −3.62, *p* = 0.004, *d* = − 0.34; Exp 2: *t*(141) = −4.65, *p* < 0.001, *d* = − 0.28]. The repeated-measures ANOVA on response times revealed an expectation by subsequent memory interaction in Experiment 2 [*F*(1,139) = 12.39, *p* < 0.001, η_p_^2^ = 0.08], with faster familiarity responses for expected stimuli [*t*(139) = −3.18, *p* = 0.01, *d* = −0.16], and faster recollection responses for unexpected stimuli [*t*(139) = 6.29, *p* < 0.001, *d* = 0.60]. This effect was not found in Experiment 1 [*F*(1,54) < 1; full results from response times are presented in the Supplementary material and means are presented in Supplementary Table 3]. Overall, there findings are consistent with the previously reported opposite effect of expectation on subsequent memory (see Introduction).

### 3.4 The effect of personality traits and individual differences

#### 3.4.1 Experiment 1: anxiety and risk-taking

##### 3.4.1.1 Risk-taking

**Figure 4:**
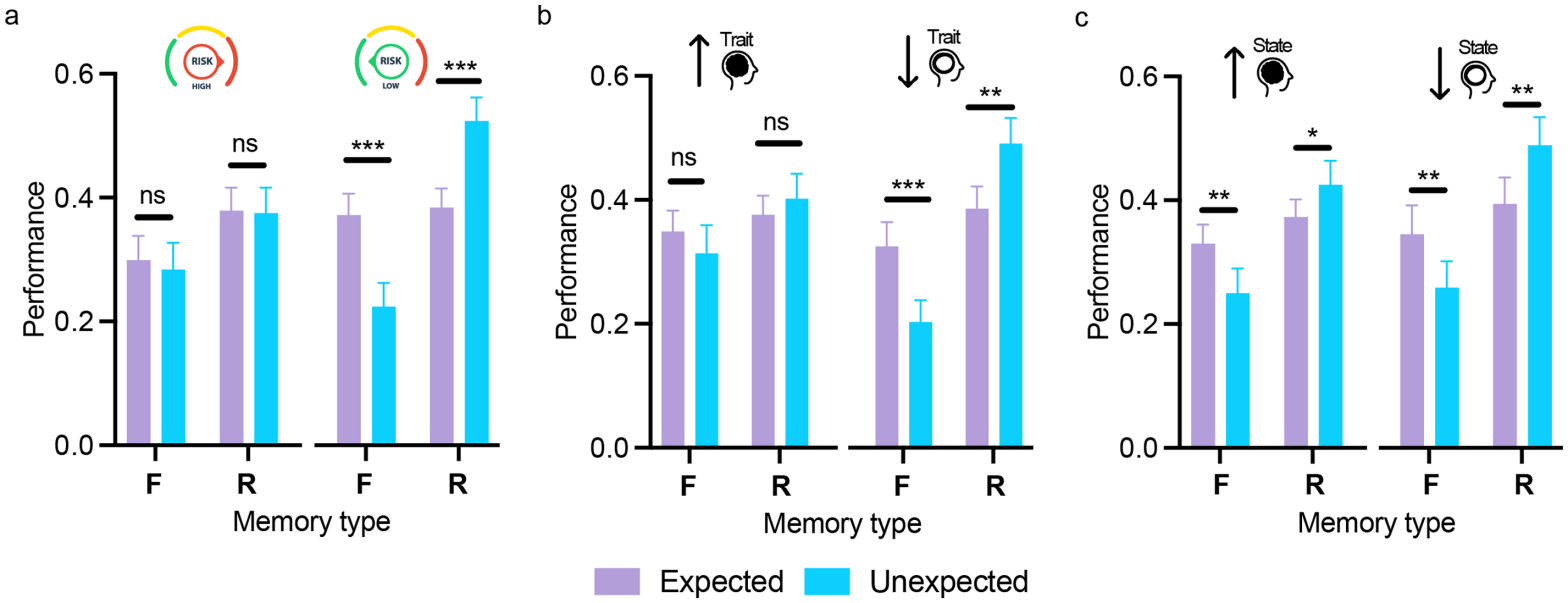
The influence of risk-taking and anxiety on expectation-modulated memory encoding. Memory performance across memory type (F/R) for expected and unexpected stimuli, shown separately for participants with high and low risk-taking behavior **(a)**, high and low trait anxiety **(b)**, and high and low state anxiety **(c)**. The differential effect of expectation on subsequent memory interacted with risk-taking and trait anxiety, emerging predominantly in participants with low levels of these traits. Error bars represent the standard error of the mean. ns = not significant; *p < 0.05; **p < 0.01; ***p < 0.001. Scatterplots displaying individual participant values across the experimental conditions for the three panels are provided in Supplementary Figure 2.

The mixed ANOVA with expectation, memory type (within-subjects) and risk-taking (high, low) on memory performance showed a significant main effect of risk-taking [*F* (1,53) = 5.54, *p = 0*.02, *η*_p_^2^ = 0.01] with higher overall memory performance in the low (*M* = 0.38, SD = 0.07) compared to the high risk-taking group (*M* = 0.33, SD = 0.07). The interactions between risk-taking and memory type or expectation were not significant (both *F*s < 1). However, the three-way interaction (expectation × memory type × risk-taking) was significant [*F*(1,53) = 18.10, *p < 0*.001, *η*_p_^2^ = 0.26], indicating strong expectation effects on performance across memory types in the low risk-taking group [familiar: *t*(53) = 6.25, *p < 0*.001, *d = 0*.75; recollected: *t*(53) = −5.92, *p < 0*.001, *d = −0*.71], but no effects in the high risk-taking group (all *t*s < 1; see Figure 4a).

##### 3.4.1.2 Trait anxiety (TA)

The mixed ANOVA with expectation, memory type (within-subjects) and trait anxiety (high, low) on memory performance showed a non-significant main effect of TA, indicating comparable memory performance between participants with high and low TA [*F*(1,53) = 0.23, *p = 0*.63, *η*_p_^2^ = 0.004]. The expectation by TA and memory type by TA interactions were also non-significant [*F*(1,53) = 0.15, *p = 0*.68, *η*_p_^2^ = 0.003 and *F*(1,53) = 0.19, *p = 0*.22, *η*_p_^2^ = 0.03, respectively]. However, the three-way interaction between expectation, memory type, and TA was significant [*F*(1,53) = 5.33, *p = 0*.025, *η*_p_^2^ = 0.09], indicating that the effect of expectation on memory performance varied by memory type and TA level. *Post hoc* tests showed opposite effects of expectation on familiarity and recollection performance in participants with low TA [expected vs. unexpected — Familiar: *t*(53) = 4.87, *p < 0*.001, *d = 0*.61; Recollected: *t*(53) = −4.22, *p = 0*.002, *d = −0*.53] but no effects in high TA (all *t <* 1; see Figure 4b). Thus, expectation effects on memory were observed only in participants with low TA.

##### 3.4.1.3 State anxiety (SA)

The mixed ANOVA with expectation, memory type (within-subjects) and state anxiety (high, low) on memory performance showed a non-significant main effect of SA [*F*(1,53) = 2.12, *p = 0*.15, *η*_p_^2^ = 0.04], indicating comparable memory performance between participants with high and low SA. The expectation by SA interaction was marginally significant [*F*(1,53) = 4.12, *p = 0*.05, *η*_p_^2^ = 0.07], suggesting that memory performance for expected and unexpected stimuli varied with SA level. Simple main effects showed better memory performance for expected versus unexpected stimuli in the high SA group (*p* = 0.022), with no difference in the low SA group (*p* = 0.53). All other interactions with SA were not significant [memory type × SA: *F*(1,53) = 0.09, *p = 0*.77, *η*_p_^2^ = 0.002; expectation x memory type × SA: *F*(1,53) = 0.41, *p = 0*.53, *η*_p_^2^ = 0.008; Figure 4c].

#### 3.4.2 Modeling trial-level prediction errors and their influence on memory

We conducted additional modeling analyses in Experiment 1 to investigate how trial-by-trial learning signals – specifically, prediction errors derived from individual learning trajectories – influence subsequent memory. Unlike condition-level analyses, this approach captures participants’ dynamic learning during the rule-learning phase and uses these personalized estimates of expectation violation to predict later memory outcomes. This method allowed us to ask whether memory is shaped not just by global manipulations of expectation, but by the moment-to-moment surprise experienced at encoding. We further examined whether this relationship is moderated by individual differences in anxiety (state and trait) and risk-taking. A reinforcement learning RW model (see Methods) was applied to participants’ trial-level responses during the rule-learning task. These responses were used to update internal associative strengths (*V*) for each symbol–category pairing (see Equation 1) and we then calculated prediction error (PE) on each learning trial as the difference between the observed outcome and the model’s current expectation (see Equation 2). To validate the model, we compared RW against a Temporal Difference (TD) model and a probabilistic Bayesian learner using AIC and BIC (RW AIC: *M* = −212.65; TD AIC: *M* = 12.31; Bayesian learner AIC: *M* = −198.82). The RW model consistently yielded better fits across participants, supporting its suitability for capturing learning in this task. While TD and Bayesian models offer richer frameworks for sequential updating, RW provided a more parsimonious fit without overfitting.

An initial two-way ANOVA on PE by recognition response (R/F/N) and expectation (expected/unexpected) revealed a marginal interaction [*F*(2, 5,115) = 2.30, *p* = 0.10]. However, this effect did not reach statistical significance and should be interpreted with caution. Logistic regression predicting recollection (R vs. F/N) showed a significant PE × expectation interaction (*B* = 0.12, *p* = 0.038) and a main effect of expectation (*B* = 0.17, *p* = 0.005), indicating that PE boosted recollection specifically for unexpected stimuli. Adding trait/state anxiety and risk-taking to the model revealed a significant three-way interaction with trait anxiety (PE × expectation × trait anxiety; *B* = −0.462, *p* = 0.009), but no significant interaction with risk-taking (*B* = −0.034, *p* = 0.81) or state anxiety (*B* = −0.031, *p* = 0.40), indicating that higher trait anxiety reduced the benefit of PE on memory under expectancy violation.

**Figure 5:**
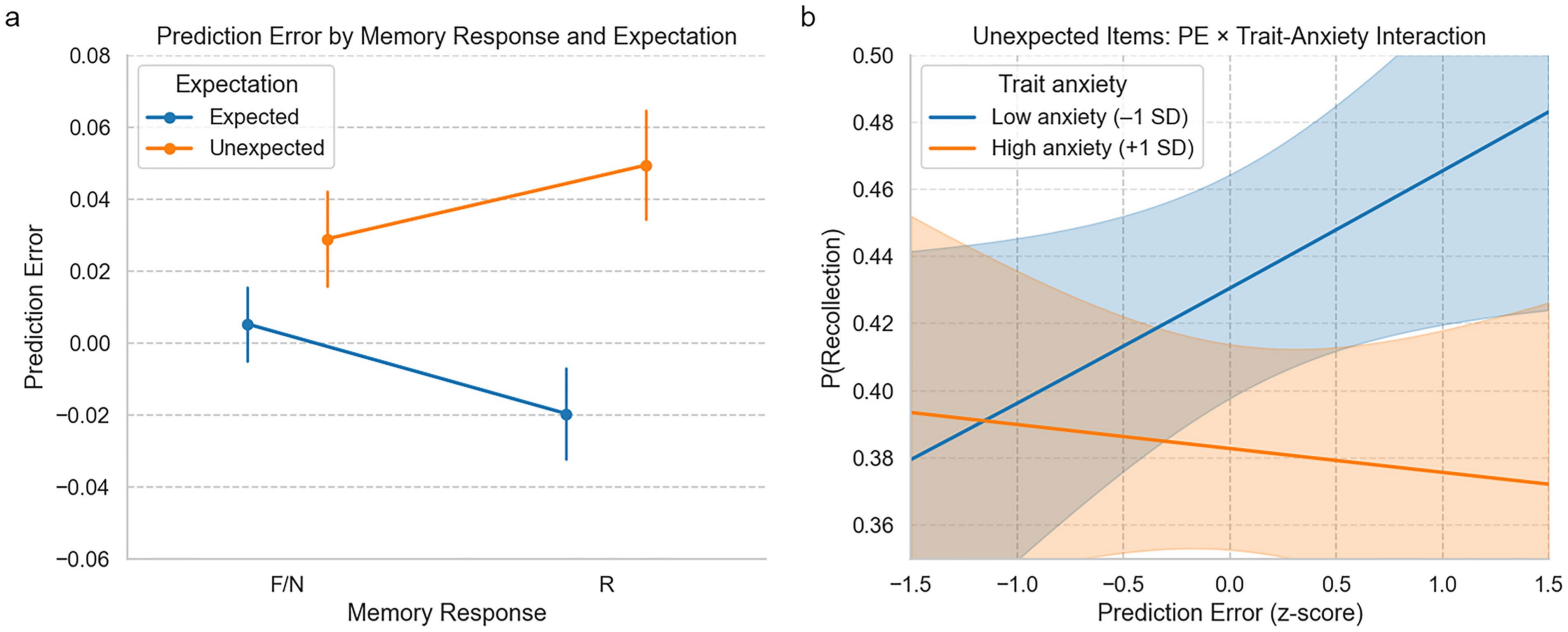
Prediction error as a function of memory response, expectation, and trait anxiety. **(a)** Average prediction error (PE) during encoding, separated by memory outcome (Recollected [R] vs. Familiar/New [F/N]) and expectation condition (expected vs. unexpected). PE was higher for subsequently recollected items than F/N responses, particularly under unexpected conditions, supporting a prediction error × expectation interaction. Error bars represent the standard error of the mean. **(b)** PE for unexpected stimuli for participants split by trait anxiety into high versus low groups. PE increased for unexpected items, but this was attenuated in the high-anxiety group, consistent with a three-way interaction (PE × Expectation × anxiety), indicating that anxiety may reduce the memory-enhancing effect of expectancy violation.

To further probe these effects, we ran a logistic regression on unexpected trials only (*n* = 1,980 trials). PE was positively but non-significantly associated with memory (*B* = 0.12, *p* = 0.30), while trait anxiety negatively predicted memory accuracy (*B* = −0.03, *p* = 0.026). Including anxiety improved model fit (McFadden’s *R*^2^ = 0.002; AIC = 2443.13). In contrast, a model with PE and risk-taking showed no significant predictors and did not outperform the null (*R*^2^ < 0.001; AIC = 2,448, *p* = 0.56). Together, these findings indicate that memory under expectancy violation is shaped by moment-to-moment individual learning signals and consistently moderated by trait anxiety (Figure 5).

#### 3.4.3 Experiment 2: personality dimensions

**Figure 6:**
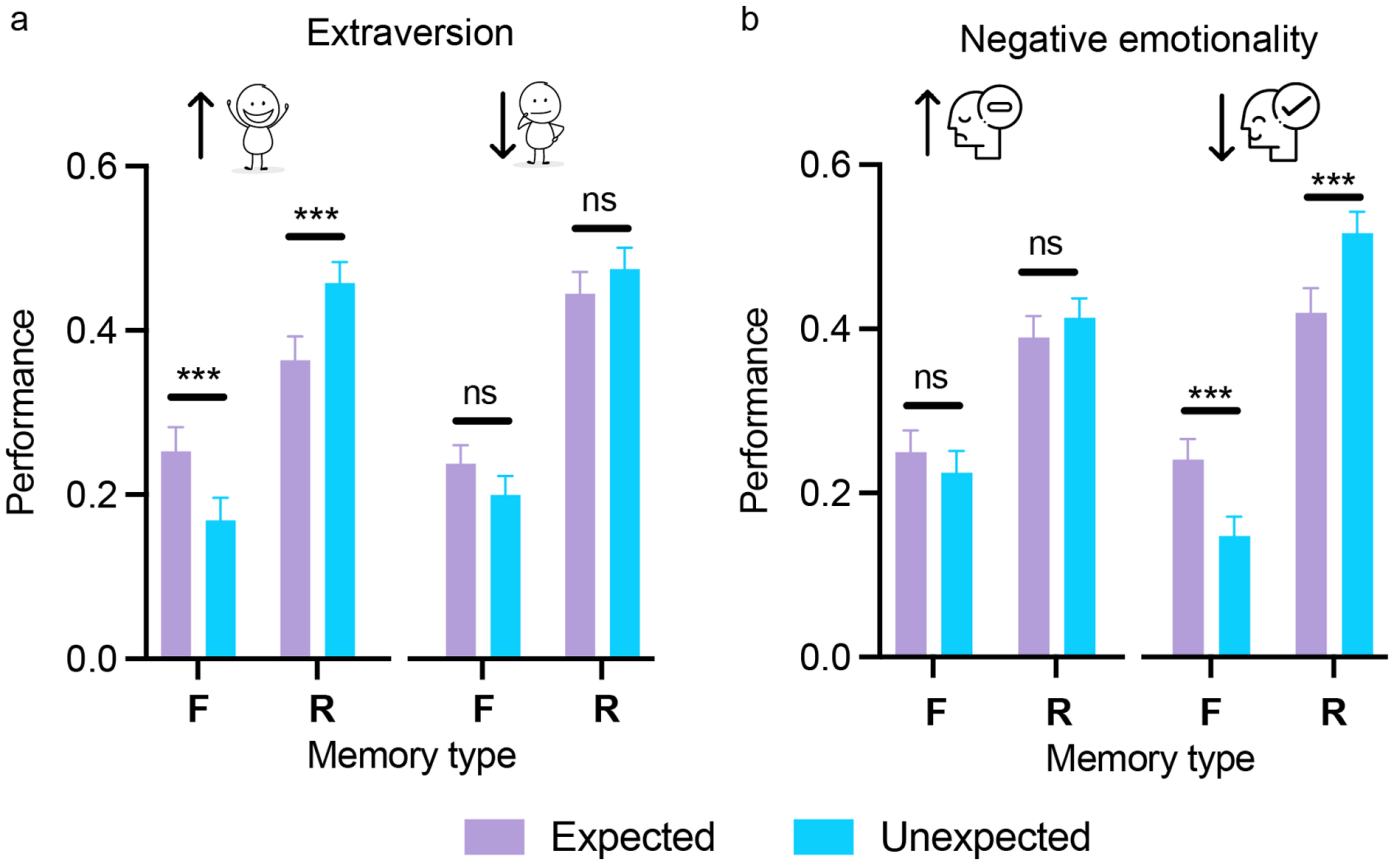
The influence of extraversion and negative emotionality on expectation-modulated memory encoding. Memory performance across memory type (F/R) for expected and unexpected stimuli, shown separately for participants with high and low extraversion **(a)** and high and low negative emotionality **(b)**. The differential effect of expectation on subsequent memory was found only in the case of participants high in extraversion and low in negative emotionality. Error bars represent the standard error of the mean. ns = not significant; ***p < 0.001. Scatterplots displaying individual participant values across the experimental conditions for the two panels are provided in Supplementary Figure 3.

Each personality dimension was included as a between-subjects factor in a three-way mixed ANOVA, alongside the two within-subjects factors (expectation and memory type) as reported in the previous sections. The analyses revealed no significant main effects of personality dimension for any of the five traits, indicating comparable overall memory performance between participants scoring high or low on each trait [extraversion: *F*(1,140) = 2.84, *p* = 0.09, *η*_p_^2^ = 0.02; agreeableness: *F*(1,140) < 1; conscientiousness: *F*(1,140) < 1; negative emotionality: *F*(1,140) < 1; open-mindedness: *F*(1,140) < 1]. Importantly, the three-way interaction between expectation, memory type, and personality domain was significant for extraversion and negative emotionality (see Table 1 for statistical outcomes), suggesting that the effect of expectation on memory varied according to levels of these traits (Figure 6). For extraversion, strong expectation-related modulation of memory performance by memory type was observed only in the high extraversion group [familiar: *t*(140) = 4.49, *p < 0*.001, *d = 0*.38; recollected: *t*(140) = −5.02, *p < 0*.001, *d = −0*.43], with no effects in the low extraversion group (all *p*s > 0.90; Figure 6a). Similarly, for negative emotionality, in the low negative emotionality group, memory for expected and unexpected stimuli diverged substantially with expected items being more likely to be recognized as familiar, whereas unexpected items were more often recollected [familiar: *t*(140) = 5.17, *p* < 0.001, *d* = 0.43; recollected: *t*(140) = −5.37, *p* < 0.001, *d* = −0.44]. In contrast, in the high negative emotionality group, no such differentiation emerged (all *p*s > 0.90), indicating that heightened negative emotionality attenuated the typical expectation-related dissociation between familiarity and recollection (Figure 6b).

**Table 1:** The effect of the five personality domains on the differential modulation of memory by expectation.

Dimension	*F* (1,140)	*p*-value	η_p_^2^
Extraversion	**4.65**	**0.03**	0.04
Agreeableness	1.76	0.19	0.01
Conscientiousness	0.77	0.38	0.01
Negative emotionality	**7.75**	**0.006**	0.05
Open mindedness	<1	0.99	0.00

The inferential statistics correspond to the expectation by memory type by personality dimension interaction. *p*-values in bold indicate significant effects; η_p_^2^ is partial-eta squared.

#### 3.4.4 Multiple regression analyses

**Figure 7:**
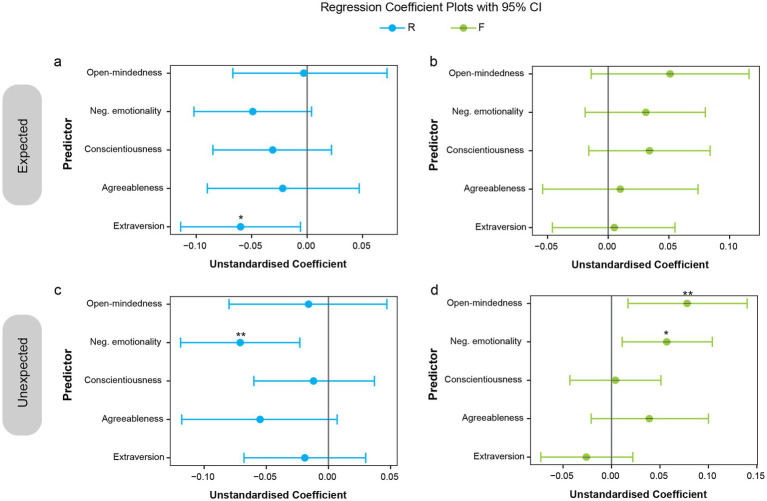
Regression coefficients (*B*) showing impact of personality traits on memory performance for expected and unexpected events. Plots of regression coefficients with 95% confidence intervals for each predictor (personality dimension), shown separately for memory performance on expected **(a,b)** and unexpected **(c,d)** stimuli. Predictors whose confidence intervals do not cross zero are statistically significant. *p < 0.05; **p < 0.01.

**Figure 8:**
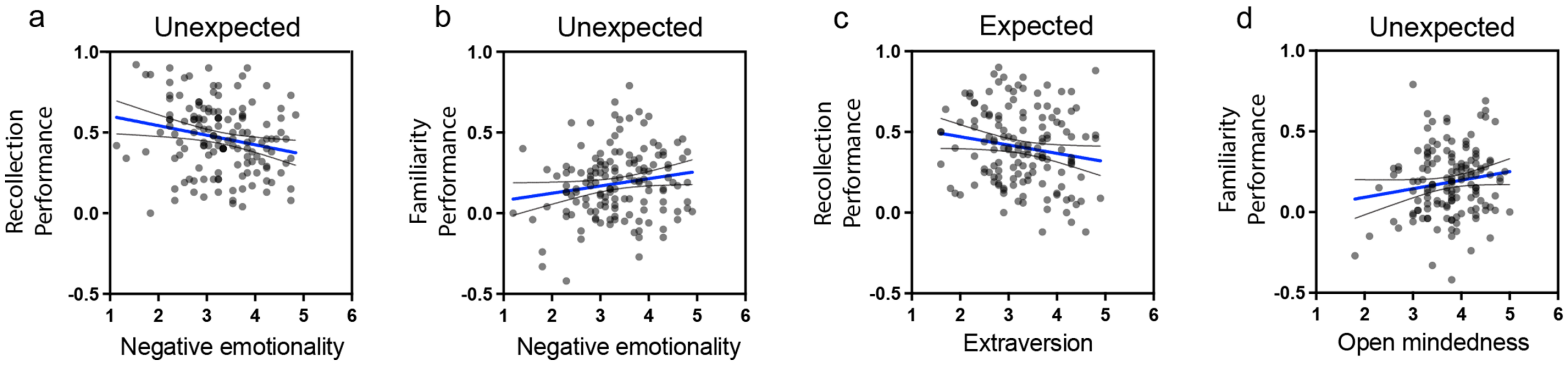
Scatterplots with regression lines depicting significant relationships between memory performance and personality traits. **(a)** negative relationship between negative emotionality and recollection performance for unexpected events, **(b)** positive relationship between negative emotionality and familiarity performance for unexpected events, **(c)** negative relationship between extraversion and familiarity performance for expected events, and **(d)** positive relationship between open-mindedness and familiarity performance for unexpected events. Scatterplots for all regressions involving personality dimensions and the four memory outcomes are presented in Supplementary Figure 4.

To further examine the influence of personality dimensions on memory performance for expected and unexpected stimuli in Experiment 2, multiple regression analyses were conducted. Memory performance, separately for recollection-based and familiarity-based performance, for each expectation condition (expected and unexpected), served as the dependent variable, with the five personality dimensions as predictors. Separate analyses were performed. Figure 7 presents the regression coefficients (*B*) with 95% confidence intervals. Significant regression plots appear in Figure 8. A summary of all coefficients is provided in Supplementary Tables 4–8, and all scatterplots with regression lines are shown in Supplementary Figure 4.

The results indicate that higher extraversion was associated with reduced recollection performance for expected stimuli (Figures 6a, 7a, 8c; *B* = −0.06, SE = 0.027, *β* = −0.195, *t*(136) = −2.19, *p* = 0.03, CI = [−0.114, −0.006]). For unexpected stimuli, higher negative emotionality was linked to reduced recollection performance (Figures 7a,c, 8a; *B* = −0.071, SE = 0.024, *β* = −0.26, *t*(136) = −2.93, *p* = 0.004, CI = [−0.119, −0.023]) but enhanced familiarity performance (Figures 7d, 8b; *B* = 0.057, SE = 0.024, *β* = 0.214, *t*(136) = 2.43, *p* = 0.02, CI = [0.011, 0.104]). Additionally, higher open-mindedness was associated with increased familiarity performance for unexpected stimuli (Figures 7d, 8d; *B* = 0.078, SE = 0.031, *β* = 0.222, *t*(136) = 2.52, *p* = 0.01, CI = [0.017, 0.14]). Overall, these findings indicate that personality traits modulate the differential effect of expectation on subsequent memory.

## 4 Discussion

In this study, we examined whether personality traits and individual differences modulate how expected and unexpected events are prioritized in memory. Across two experiments, we replicated the previously documented expectation effect on memory (Kafkas and Montaldi, 2018a; Kafkas, 2021). We predicted that expected stimuli would enhance familiarity-based recognition, whereas unexpected stimuli would preferentially enhance recollection. Consistent with this, conforming (rule-consistent) stimuli tended to be remembered via familiarity, whereas violating (rule-inconsistent) stimuli yielded richer associative recollection. We further hypothesized that high levels of anxiety and negative emotionality would attenuate the recollection boost for unexpected stimuli, whereas higher risk-taking and extraversion would enhance it, reflecting contrasting motivational orientations toward uncertainty and novelty (Zsido et al., 2020; Blais and Weber, 2006; Smillie et al., 2019). The results partly supported these predictions. Participants with lower trait anxiety and lower risk-taking showed a more pronounced recollection advantage for unexpected stimuli, whereas those high in these traits did not differentiate strongly between expected and unexpected events. The effect of trait anxiety was also robust when considering trial-specific prediction errors and individual learning trajectories. In Experiment 2, personality dimensions, particularly extraversion and negative emotionality, further qualified the expectation–memory relationship. Low extraversion and high negative emotionality reduced the recollection advantage for unexpected events, again in line with our predictions for affective traits but not for extraversion. These findings confirm that the mechanisms through which novelty or prediction errors enhance memory are shaped, and sometimes constrained, by broader individual differences related to the way people engage with their environment.

These findings align with theoretical accounts proposing that expectation violations activate adaptive learning processes, driving exploratory behaviors and updating mental representations (de Lange et al., 2018; Foster and Keane, 2019; Frank and Kafkas, 2021; Kafkas and Montaldi, 2018b; Quent et al., 2021). While unexpected information facilitates flexible memory formation through enriched encoding, the extent to which this benefit is realized depends fundamentally on dispositional traits influencing engagement with the environment. High trait anxiety and negative emotionality may attenuate recollection benefits for unexpected stimuli by promoting threat-related vigilance, self-focused attention, or avoidance behaviors (Zsido et al., 2020; Soto and John, 2017). Neuroimaging evidence indicates that anxiety increases amygdala–hippocampus connectivity during emotional encoding (Qasim et al., 2023), potentially diverting processing resources away from novel or surprising input. As a result, individuals high in anxiety or negative emotionality may exhibit diminished memory for unexpected events, with heightened amygdala activity biasing hippocampal encoding toward expected stimuli instead (Zhang et al., 2020; Zsido et al., 2020). This pattern agrees with computational models (Pulcu and Browning, 2017; Yu and Dayan, 2005), which posit that an intolerance of uncertainty (Carleton et al., 2012) can lead individuals to down-weight surprising information, thereby impairing adaptive encoding. That is, when all events are experienced in a heightened emotional state, the mnemonic advantage of unexpected events can be diminished, as was the case in the present findings. Finally, high *state* anxiety showed a marginal association with greater overall memory for expected stimuli (*p* = 0.05), suggesting that transient affective states may modulate the expectation effect on memory, though this trend should be interpreted with caution.

Contrary to our hypothesis, that high risk-taking would lead to enhanced recollection for unexpected stimuli, we found comparable memory performance for expected and unexpected stimuli, while the recollection advantage of unexpected stimuli was retained in low risk-taking individuals. Although previous research suggests that risk-takers may effectively orient attention toward novel stimuli (Huo et al., 2020; Schneider et al., 2012), our findings indicate this attentional orientation might not translate into superior memory encoding. Notably, the condition-level analysis revealed that the recollection benefit from expectancy violation was specific to individuals low in risk-taking. One plausible explanation is that the impulsivity and sensation-seeking traits associated with risk-taking lead to more superficial or inconsistent encoding, particularly in response to expectancy violations (Bechara, 2000; Zuckerman, 2007). Rather than allocating sustained cognitive effort to encode surprising information, high risk-takers may rely on more spontaneous, less deliberative strategies (Kahneman, 2011; Kirchler et al., 2017), resulting in reduced task engagement and diminished recollection.

In contrast, our modeling analysis, which examined trial-by-trial learning signals (i.e., prediction errors), did not reveal any modulation by risk-taking. This difference may suggest that while risk-taking may influence overall task engagement or strategic encoding, it does not appear to affect the moment-to-moment processing of expectancy violations. Unlike trait anxiety, risk-taking may thus exert a more global (task-wide) rather than dynamic (trial-by-trial) influence on memory performance. In other words, risk-taking may influence memory at a broader strategic level – consistent with the condition-level effect, rather than at the level of trial-by-trial learning signals.

Extraversion also modulated the expectation effect on recollection, but in a distinct manner: higher extraversion was associated with reduced recollection for expected stimuli. Consequently, the differential effect of expectation on recollection was more pronounced among individuals with high extraversion and diminished among those with low extraversion. This result may reflect extraverts’ preference for broad, gist-based encoding rather than detailed, item-specific encoding strategies, particularly when stimuli align predictably with existing knowledge (DeYoung, 2013; Smillie et al., 2019). Indeed, extraverts allocate attention toward emotionally or socially salient aspects of experiences, potentially at the expense of encoding routine details (Smillie, 2013). Thus, extraverts’ natural tendency toward external engagement and reward-seeking behavior may shift cognitive resources away from detailed processing of the more mundane, expected events.

In relation to personality theories, these findings align with trait-activation models (Tett and Burnett, 2003), which propose that personality traits influence cognition most strongly when situational cues activate trait-relevant processes. Traits become salient in environments that elicit behaviors associated with those traits (Judge and Zapata, 2015; Van Knippenberg and Hirst, 2020). In the present study, expectation-violating events may more strongly engage traits related to novelty seeking or vigilance leading to individualized encoding strategies. Similarly, the present findings are consistent with cognitive-affective models of personality, such as the Cognitive-Affective Personality System (Mischel and Shoda, 1995), which views traits as context-dependent patterns of cognitive-affective activation. Expectation violations may serve as such trigger, differentially engaging traits like anxiety or risk tolerance. Finally, the findings suggest a novel interface between personality theory and recognition memory models, showing that personality can modulate the engagement of hippocampal versus non-hippocampal memory processes—an interaction largely overlooked in memory research to date.

Educationally, our results reinforce the practical value of presenting learners with both expected and unexpected information, leveraging complementary encoding mechanisms that result in different memory experiences (Kafkas, 2021; Kafkas and Montaldi, 2018a). However, acknowledging individual differences, particularly anxiety and risk-taking, is crucial. Specifically, given that higher anxiety was associated with poorer memory for unexpected events, anxious learners might benefit more from structured and predictable content that minimizes uncertainty during learning. This finding is consistent with evidence that anxiety is characterized by intolerance of uncertainty (Carleton, 2012) and attentional avoidance of unpredictability (Grupe and Nitschke, 2013). Individuals high in anxiety often perceive uncertainty as aversive and engage in cognitive and emotional strategies to reduce exposure to ambiguous or surprising outcomes (Topel et al., 2024; White et al., 2017). Accordingly, we infer that educational settings with high predictability—where expectations are more often confirmed than violated—could reduce cognitive load and facilitate more effective memory in anxious learners. In contrast, highly extraverted or risk-seeking learners may require targeted strategies that increase engagement to enhance detailed precision-weighted encoding of information.

Taken together, the present findings extend predictive-processing accounts of memory by showing that personality traits modulate how individuals respond to expectancy violations. Traits such as anxiety and risk-taking may alter the precision weighting of prediction errors, thereby influencing whether unexpected events trigger enhanced recollection or disrupted encoding. This framework suggests that individual differences in motivational and affective orientation shape the dynamic balance between exploiting predictable regularities and exploring novel information — a mechanism that can account for the diverse effects of expectation on memory observed across participants.

The current study relied on self-report measures and relatively homogeneous samples. Future research employing larger, more diverse populations and combining self-report with behavioral or neurobiological measures could enrich understanding of these phenomena. A further limitation concerns the gender composition of our samples. Experiment 1 included predominantly female participants and Experiment 2, although larger, remained unbalanced. As some personality traits such as anxiety, risk-taking, and extraversion show modest gender differences (e.g., Costa et al., 2001; Cross and Madson, 1997), this imbalance could have influenced the magnitude of the observed personality effects. However, similar expectation-modulated memory patterns were found across both experiments, suggesting that the core effects are unlikely to be solely driven by gender. Future studies should employ more gender-balanced samples.

Future research is also required to also investigate clinical populations, such as individuals with depression, to extend our understanding of expectation-modulated memory processes. Indeed, in our study, participants’ mean trait anxiety scores were comparable to those reported in non-clinical samples (Zsido et al., 2020), whereas mean state anxiety scores were slightly lower. This suggests that the present findings primarily reflect variability within the normal range of anxiety rather than clinical levels. Nevertheless, it is plausible that clinically elevated anxiety would further attenuate memory benefits for unexpected stimuli, given evidence that high anxiety narrows attentional focus and increases avoidance of uncertainty (Bishop, 2009; Robinson et al., 2013). Such effects could exaggerate the dampening of recollection we observed in more anxious participants. Therefore, future work including clinically anxious samples would be valuable for testing whether the modulation of expectation-based memory generalizes beyond typical population ranges. Additionally, given that anxiety can be characterized as an increased sensitivity to uncertainty, exploring interventions tailored to such specific personality traits, such as mindfulness training for anxious individuals, may further illustrate the flexible nature of expectation-modulated memory processes (Schaeffer et al., 2021).

## 5 Conclusion

In conclusion, the present findings significantly extend our understanding of how expectation violations and prediction errors interact with individual differences to shape memory. While unexpected events commonly facilitate detailed episodic recollection, the consistency and magnitude of this effect are shaped by one’s personality traits related to the way they engage with the environment. The current results underscore that memory processes are neither fixed nor uniform but interact dynamically with dispositional factors such as anxiety, risk-taking, extraversion, and negative emotionality. Understanding these interactions offers promising avenues for refining cognitive and applied models of memory.

## Data Availability

The datasets presented in this study can be found in online repositories. The names of the repository/repositories and accession number(s) can be found below: Open Science Framework repository: https://osf.io/hnx5s/?view_only=6d8bc56c6f324795ba3dd187c9fc2f2d.

## Footnotes

1
https://gorilla.sc/

## References

[ref1] Ausmees L. Realo A. Allik J. (2022). Episodic memory reliving and personality: do good *time travelers* have distinctive personality profiles? J. Individ. Differ. 43, 47–54. doi: 10.1027/1614-0001/a000353

[ref2] Bar M. (2009). The proactive brain: memory for predictions. Philos. Trans. R. Soc. Lond. Ser. B Biol. Sci. 364, 1235–1243. doi: 10.1098/rstb.2008.0310, 19528004 PMC2666710

[ref3] Barkasi M. Rosen M. G. (2020). Is mental time travel real time travel? Philos. Mind Sci. 1, 1–27. doi: 10.33735/phimisci.2020.1.28

[ref4] Bechara A. (2000). Emotion, decision making and the orbitofrontal cortex. Cereb. Cortex 10, 295–307. doi: 10.1093/cercor/10.3.295, 10731224

[ref5] Bein O. Livneh N. Reggev N. Gilead M. Goshen-Gottstein Y. Maril A. (2015). Delineating the effect of semantic congruency on episodic memory: the role of integration and relatedness. PLoS One 10:e0115624. doi: 10.1371/journal.pone.0115624, 25695759 PMC4335002

[ref6] Bishop S. J. (2009). Trait anxiety and impoverished prefrontal control of attention. Nat. Neurosci. 12, 92–98. doi: 10.1038/nn.2242, 19079249

[ref7] Blais A.-R. Weber E. U. (2006). A domain-specific risk-taking (DOSPERT) scale for adult populations. Judgm. Decis. Mak. 1, 33–47. doi: 10.1017/S1930297500000334

[ref8] Bollinger J. Rubens M. T. Zanto T. P. Gazzaley A. (2010). Expectation-driven changes in cortical functional connectivity influence working memory and long-term memory performance. J. Neurosci. 30, 14399–14410. doi: 10.1523/JNEUROSCI.1547-10.2010, 20980597 PMC3006446

[ref9] Carleton R. N. (2012). The intolerance of uncertainty construct in the context of anxiety disorders: theoretical and practical perspectives. Expert. Rev. Neurother. 12, 937–947. doi: 10.1586/ern.12.82, 23002938

[ref10] Carleton R. N. Mulvogue M. K. Thibodeau M. A. McCabe R. E. Antony M. M. Asmundson G. J. G. (2012). Increasingly certain about uncertainty: intolerance of uncertainty across anxiety and depression. J. Anxiety Disord. 26, 468–479. doi: 10.1016/j.janxdis.2012.01.011, 22366534

[ref11] Clos M. Bunzeck N. Sommer T. (2019). Dopamine enhances item novelty detection via hippocampal and associative recall via left lateral prefrontal cortex mechanisms. J. Neurosci. 39, 7920–7933. doi: 10.1523/JNEUROSCI.0495-19.2019, 31405927 PMC6774414

[ref12] Costa P. T. Terracciano A. McCrae R. R. (2001). Gender differences in personality traits across cultures: robust and surprising findings. J. Pers. Soc. Psychol. 81, 322–331. doi: 10.1037/0022-3514.81.2.322, 11519935

[ref13] Cross S. E. Madson L. (1997). Models of the self: self-construals and gender. Psychol. Bull. 122, 5–37. doi: 10.1037/0033-2909.122.1.5, 9204777

[ref14] de Lange F. P. Heilbron M. Kok P. (2018). How do expectations shape perception? Trends Cogn. Sci. 22, 764–779. doi: 10.1016/j.tics.2018.06.002, 30122170

[ref15] De Loof E. Ergo K. Naert L. Janssens C. Talsma D. Van Opstal F. . (2018). Signed reward prediction errors drive declarative learning. PLoS One 13:e0189212. doi: 10.1371/journal.pone.0189212, 29293493 PMC5749691

[ref16] DeYoung C. G. (2013). The neuromodulator of exploration: a unifying theory of the role of dopamine in personality. Front. Hum. Neurosci. 7:00762. doi: 10.3389/fnhum.2013.00762, 24294198 PMC3827581

[ref17] Eysenck H. J. (1981). A model for personality. Berlin, Heidelberg: Springer.

[ref18] Figner B. Weber E. U. (2011). Who takes risks when and why?: determinants of risk taking. Curr. Dir. Psychol. Sci. 20, 211–216. doi: 10.1177/0963721411415790

[ref19] Foster M. I. Keane M. T. (2019). The role of surprise in learning: different surprising outcomes affect memorability differentially. Top. Cogn. Sci. 11, 75–87. doi: 10.1111/tops.12392, 30375159

[ref20] Frank D. Kafkas A. (2021). Expectation-driven novelty effects in episodic memory. Neurobiol. Learn. Mem. 183:107466. doi: 10.1016/j.nlm.2021.107466, 34048914

[ref21] Frank D. Montemurro M. A. Montaldi D. (2020). Pattern separation underpins expectation-modulated memory. J. Neurosci. 40, 3455–3464. doi: 10.1523/JNEUROSCI.2047-19.2020, 32161140 PMC7178906

[ref22] Friston K. (2010). The free-energy principle: a unified brain theory? Nat. Rev. Neurosci. 11, 127–138. doi: 10.1038/nrn2787, 20068583

[ref23] Greve A. Cooper E. Tibon R. Henson R. N. (2019). Knowledge is power: prior knowledge aids memory for both congruent and incongruent events, but in different ways. J. Exp. Psychol. Gen. 148, 325–341. doi: 10.1037/xge0000498, 30394766 PMC6390882

[ref24] Grupe D. W. Nitschke J. B. (2013). Uncertainty and anticipation in anxiety: an integrated neurobiological and psychological perspective. Nat. Rev. Neurosci. 14, 488–501. doi: 10.1038/nrn3524, 23783199 PMC4276319

[ref25] Huo H. Zhang R. Seger C. A. Feng T. Chen Q. (2020). The effect of trait anxiety on risk-taking: functional coupling between right hippocampus and left insula. Psychophysiology 57:e13629. doi: 10.1111/psyp.13629, 32628786

[ref26] Jang A. I. Nassar M. R. Dillon D. G. Frank M. J. (2019). Positive reward prediction errors during decision-making strengthen memory encoding. Nat. Hum. Behav. 3, 719–732. doi: 10.1038/s41562-019-0597-3, 31061490 PMC6625913

[ref27] Judge T. A. Zapata C. P. (2015). The person–situation debate revisited: effect of situation strength and trait activation on the validity of the big five personality traits in predicting job performance. Acad. Manag. J. 58, 1149–1179. doi: 10.5465/amj.2010.0837

[ref28] Kafkas A. (2021). Encoding-linked pupil response is modulated by expected and unexpected novelty: implications for memory formation and neurotransmission. Neurobiol. Learn. Mem. 180:107412. doi: 10.1016/j.nlm.2021.107412, 33609740

[ref29] Kafkas A. Montaldi D. (2015). Striatal and midbrain connectivity with the hippocampus selectively boosts memory for contextual novelty. Hippocampus 25, 1262–1273. doi: 10.1002/hipo.22434, 25708843 PMC4672698

[ref30] Kafkas A. Montaldi D. (2018a). Expectation affects learning and modulates memory experience at retrieval. Cognition 180, 123–134. doi: 10.1016/j.cognition.2018.07.010, 30053569 PMC6191926

[ref31] Kafkas A. Montaldi D. (2018b). How do memory systems detect and respond to novelty? Neurosci. Lett. 680, 60–68. doi: 10.1016/j.neulet.2018.01.053, 29408218 PMC6565889

[ref32] Kahneman D. (2011). Thinking, fast and slow. London: Allen Lane.

[ref33] Kirchler M. Andersson D. Bonn C. Johannesson M. Sørensen E. Ø. Stefan M. . (2017). The effect of fast and slow decisions on risk taking. J. Risk Uncertain. 54, 37–59. doi: 10.1007/s11166-017-9252-4, 28725117 PMC5486903

[ref34] Klaming R. Veltman D. J. Comijs H. C. (2017). The impact of personality on memory function in older adults—results from the longitudinal aging study Amsterdam. Int. J. Geriatr. Psychiatry 32, 798–804. doi: 10.1002/gps.4527, 27329835

[ref35] Liu Z.-X. Grady C. Moscovitch M. (2018). The effect of prior knowledge on post-encoding brain connectivity and its relation to subsequent memory. NeuroImage 167, 211–223. doi: 10.1016/j.neuroimage.2017.11.032, 29158201

[ref36] Miendlarzewska E. A. Bavelier D. Schwartz S. (2016). Influence of reward motivation on human declarative memory. Neurosci. Biobehav. Rev. 61, 156–176. doi: 10.1016/j.neubiorev.2015.11.015, 26657967

[ref37] Mischel W. Shoda Y. (1995). A cognitive-affective system theory of personality: Reconceptualizing situations, dispositions, dynamics, and invariance in personality structure. Psychol. Rev. 102, 246–268. doi: 10.1037/0033-295X.102.2.246, 7740090

[ref38] Montaldi D. Kafkas A. (2024). “Neural mechanisms of familiarity” in The Oxford handbook of human memory: Foundations and applications. eds. Wagner A. D. Kahana M. J. (USA: Oxford University Press).

[ref39] Press C. Yon D. (2019). Perceptual prediction: rapidly making sense of a noisy world. Curr. Biol. 29, R751–R753. doi: 10.1016/j.cub.2019.06.054, 31386853

[ref40] Pulcu E. Browning M. (2017). Affective bias as a rational response to the statistics of rewards and punishments. eLife 6:e27879. doi: 10.7554/eLife.27879, 28976304 PMC5633345

[ref41] Qasim S. E. Mohan U. R. Stein J. M. Jacobs J. (2023). Neuronal activity in the human amygdala and hippocampus enhances emotional memory encoding. Nat. Hum. Behav. 7, 754–764. doi: 10.1038/s41562-022-01502-8, 36646837 PMC11243592

[ref42] Quent J. A. Henson R. N. Greve A. (2021). A predictive account of how novelty influences declarative memory. Neurobiol. Learn. Mem. 179:107382. doi: 10.1016/j.nlm.2021.107382, 33476747 PMC8024513

[ref43] Reichardt R. Polner B. Simor P. (2020). Novelty manipulations, memory performance, and predictive coding: the role of unexpectedness. Front. Hum. Neurosci. 14:152. doi: 10.3389/fnhum.2020.00152, 32410975 PMC7201021

[ref44] Robinson M. D. (2004). Personality as performance: categorization tendencies and their correlates. Curr. Dir. Psychol. Sci. 13, 127–129. doi: 10.1111/j.0963-7214.2004.00290.x

[ref45] Robinson O. J. Vytal K. Cornwell B. R. Grillon C. (2013). The impact of anxiety upon cognition: perspectives from human threat of shock studies. Front. Hum. Neurosci. 7:203. doi: 10.3389/fnhum.2013.00203, 23730279 PMC3656338

[ref46] Schaeffer J. D. Newell C. Spann C. Siemens G. Liegey Dougall A. (2021). Inflammation, depression, and anxiety related to recognition memory in young adults. J. Gen. Psychol. 150, 1–25. doi: 10.1080/00221309.2021.1893638, 33729100

[ref47] Schmidt S. R. Schmidt C. R. (2017). Revisiting von Restorff’s early isolation effect. Mem. Cogn. 45, 194–207. doi: 10.3758/s13421-016-0651-6, 27631791

[ref48] Schneider S. Peters J. Bromberg U. Brassen S. Miedl S. F. Banaschewski T. . (2012). Risk taking and the adolescent reward system: a potential common link to substance abuse. Am. J. Psychiatry 169, 39–46. doi: 10.1176/appi.ajp.2011.11030489, 21955931

[ref49] Smillie L. D. (2013). Extraversion and reward processing. Curr. Dir. Psychol. Sci. 22, 167–172. doi: 10.1177/0963721412470133 PMC3989147 24748727

[ref50] Smillie L. D. Jach H. K. Hughes D. M. Wacker J. Cooper A. J. Pickering A. D. (2019). Extraversion and reward-processing: consolidating evidence from an electroencephalographic index of reward-prediction-error. Biol. Psychol. 146:107735. doi: 10.1016/j.biopsycho.2019.107735, 31352030

[ref51] Soto C. J. John O. P. (2017). The next big five inventory (BFI-2): developing and assessing a hierarchical model with 15 facets to enhance bandwidth, fidelity, and predictive power. J. Pers. Soc. Psychol. 113, 117–143. doi: 10.1037/pspp0000096, 27055049

[ref9001] Spielberger C. D. Gorsuch R. L. Lushene R. Vagg P. R. Jacobs G. A. (1983). Manual for the State-Trait Anxiety Inventory. Palo Alto, CA: Consulting Psychologists Press.

[ref52] Stolz C. Bulla A. Soch J. Schott B. H. Richter A. (2023). Openness to experience is associated with neural and performance measures of memory in older adults. Soc. Cogn. Affect. Neurosci. 18:nsad041. doi: 10.1093/scan/nsad041, 37632761 PMC10533339

[ref53] Tett R. P. Burnett D. D. (2003). A personality trait-based interactionist model of job performance. J. Appl. Psychol. 88, 500–517. doi: 10.1037/0021-9010.88.3.500, 12814298

[ref54] Topel S. Ma I. Van Duijvenvoorde A. C. K. Van Steenbergen H. De Bruijn E. R. A. (2024). Adapting to uncertainty: the role of anxiety and fear of negative evaluation in learning in social and non-social contexts. J. Affect. Disord. 363, 310–319. doi: 10.1016/j.jad.2024.07.066, 39043306

[ref55] Van Der Wel P. Van Steenbergen H. (2018). Pupil dilation as an index of effort in cognitive control tasks: a review. Psychon. Bull. Rev. 25, 2005–2015. doi: 10.3758/s13423-018-1432-y, 29435963 PMC6267528

[ref56] Van Kesteren M. T. R. Krabbendam L. Meeter M. (2018). Integrating educational knowledge: reactivation of prior knowledge during educational learning enhances memory integration. NPJ Sci. Learn. 3:11. doi: 10.1038/s41539-018-0027-8, 30631472 PMC6220240

[ref57] Van Knippenberg D. Hirst G. (2020). A motivational lens model of person × situation interactions in employee creativity. J. Appl. Psychol. 105, 1129–1144. doi: 10.1037/apl0000486, 31985236

[ref58] Weber E. U. Blais A.-R. Betz N. E. (2002). A domain-specific risk-attitude scale: measuring risk perceptions and risk behaviors. J. Behav. Decis. Mak. 15, 263–290. doi: 10.1002/bdm.414

[ref59] Weller J. Ceschi A. Hirsch L. Sartori R. Costantini A. (2018). Accounting for individual differences in decision-making competence: personality and gender differences. Front. Psychol. 9:2258. doi: 10.3389/fpsyg.2018.02258, 30534098 PMC6276324

[ref60] White S. F. Geraci M. Lewis E. Leshin J. Teng C. Averbeck B. . (2017). Prediction error representation in individuals with generalized anxiety disorder during passive avoidance. Am. J. Psychiatry 174, 110–117. doi: 10.1176/appi.ajp.2016.15111410, 27631963 PMC5572647

[ref61] Wittmann B. C. Schott B. H. Guderian S. Frey J. U. Heinze H.-J. Düzel E. (2005). Reward-related FMRI activation of dopaminergic midbrain is associated with enhanced hippocampus-dependent long-term memory formation. Neuron 45, 459–467. doi: 10.1016/j.neuron.2005.01.010, 15694331

[ref62] Yau J. O.-Y. McNally G. P. (2023). The Rescorla-Wagner model, prediction error, and fear learning. Neurobiol. Learn. Mem. 203:107799. doi: 10.1016/j.nlm.2023.107799, 37442411

[ref63] Yonelinas A. P. Aly M. Wang W.-C. Koen J. D. (2010). Recollection and familiarity: examining controversial assumptions and new directions. Hippocampus 20, 1178–1194. doi: 10.1002/hipo.20864, 20848606 PMC4251874

[ref64] Yu A. J. Dayan P. (2005). Uncertainty, neuromodulation, and attention. Neuron 46, 681–692. doi: 10.1016/j.neuron.2005.04.026, 15944135

[ref65] Zhang X. Kim J. Tonegawa S. (2020). Amygdala reward neurons form and store fear extinction memory. Neuron 105, 1077–1093.e7. doi: 10.1016/j.neuron.2019.12.025, 31952856

[ref66] Zsido A. N. Teleki S. A. Csokasi K. Rozsa S. Bandi S. A. (2020). Development of the short version of the Spielberger state—trait anxiety inventory. Psychiatry Res. 291:113223. doi: 10.1016/j.psychres.2020.113223, 32563747

[ref67] Zuckerman M. (2007). Sensation seeking and risky behavior. Washington: American Psychological Association.

