# Supplementary material for: When personality meets surprise: individual differences in memory for unexpected events
Source: Front Psychol. 2025 Dec 8;16:1652428. doi: 10.3389/fpsyg.2025.1652428 (PMC12719420; doi:10.3389/fpsyg.2025.1652428)
Supplement: Supplementary file 1 [file Table_1.docx]

***Supplementary Material***

1. **Supplementary analysis on response times at recognition**

Mean response times at recognition for expected and unexpected stimuli appear in Supplementary Table 3. The ANOVAs indicated matched response times between expected and unexpected stimuli (main effect of expectation; Exp 1: *F*(1,54) = .86, *p* = .36, η_p_^2^ = .02; Exp 2: *F*(1,139) = 1.34, *p* = .25, η_p_^2^ = .01). However, recollected stimuli received faster response times than familiar ones (main effect of memory type; Exp 1: *F*(1,54) = 8.90, *p* = .004, η_p_^2^ = .14; Exp 2: *F*(1,139) = 40.42, *p* < .001, η_p_^2^ = .22). In Experiment 1, this difference was not modulated by expectation, as shown by the non-significant expectation by memory type interaction (*F*(1,54) = .10, *p* = .75, η_p_^2^ = .002). In contrast, Experiment 2 revealed a significant interaction (*F*(1,139) = 12.39, *p* < .001, η_p_^2^ = .08), showing that response times were significantly faster for expected compared to unexpected stimuli when familiarity was the basis of recognition (*t*(139) = -3.18, *p* = .01, *d* = -.16). Conversely, when recognition was based on recollection, response times were significantly faster for unexpected compared to expected stimuli (*t*(139) = 6.29, *p* < .001, *d* = 0.60; see Supplementary Table 3).

1. **Supplementary Tables**

**Supplementary Table 1.** Mean proportion of pleasantness responses across to expected and unexpected stimuli at encoding in Experiments 1 and 2

|  | **Experiment 1** | | **Experiment 2** | |
| --- | --- | --- | --- | --- |
|  | **Expected** | **Unexpected** | **Expected** | **Unexpected** |
| **Unpleasant** | 0.28 (0.14) | 0.25 (0.16) | 0.20 (0.13) | 0.20 (0.16) |
| **Neutral** | 0.32 (0.21) | 0.33 (0.22) | 0.38 (0.20) | 0.37 (0.20) |
| **Pleasant** | 0.34 (0.16) | 0.36 (0.16) | 0.37 (0.17) | 0.36 (0.16) |

*Note.* Numbers in the parentheses are standard deviations.

**Supplementary Table 2.** Proportions of recognition memory outcomes for expected and unexpected stimuli in Experiments 1 and 2

|  | **Experiment 1** | | **Experiment 2** | |
| --- | --- | --- | --- | --- |
|  | **Expected** | **Unexpected** | **Expected** | **Unexpected** |
| **Hits** | 0.85 (0.11) | 0.84 (0.12) | 0.84 (0.12) | 0.84 (0.12) |
| **Hits_F_** | 0.44 (0.18) | 0.37 (0.20) | 0.39 (0.20) | 0.33 (0.18) |
| **Hits_R_** | 0.41 (0.20) | 0.48 (0.23) | 0.45 (0.23) | 0.51 (0.21) |
| **CR** | 0.85 (0.09) | | 0.80 (0.17) | |
| **M** | 0.14 (0.11) | 0.15 (0.11) | 0.15 (0.12) | 0.15 (0.12) |
| **FA** | 0.14 (0.09) | | 0.19 (0.15) | |
| **FA_F_** | 0.12 (0.07) | | 0.14 (0.12) | |
| **FA_R_** | 0.03 (0.06) | | 0.04 (0.07) | |

*Note:* Numbers in the parentheses are standard deviations. Hits_F_ = familiarity hits; Hits_R_ = recollection hits; FA_F_ = familiarity false alarms; FA_R_ = recollection false alarms; CR = correct rejections; M = misses.

**Supplementary Table 3.** Mean response times (and standard deviations) during recognition for expected and unexpected stimuli in the two experiments

|  | **Experiment 1** | | **Experiment 2** | |
| --- | --- | --- | --- | --- |
|  | **Expected** | **Unexpected** | **Expected** | **Unexpected** |
| **Hits_F_** | 1356.81 (328.01) | 1362.23 (333.77) | 1455.20 (331.07) | 1503.81 (369.17) |
| **Hits_R_** | 1225.76 (271.83) | 1244.92 (312.00) | 1322.84 (269.96) | 1298.69 (262.29) |
| **CR** | 1166.56 (229.75) | | 1333.98 (246.79) | |
| **M** | 1310.61 (473.22) | 1256.92 (315.41) | 1479 (360.70) | 1475.83 (414.46) |
| **FA_F_** | 1376.29 (389.09) | | 1546.92 (446.20) | |
| **FA_R_** | 1400.49 (674.05) | | 1318.12 (440.09) | |

*Note:* Hits_F_ = familiarity hits; Hits_R_ = recollection hits; FA_F_ = familiarity false alarms; FA_R_ = recollection false alarms; CR = correct rejections; M = misses.

**Supplementary Table 4.** Multiple regression coefficients and model fit for predicting overall memory performance from the big five personality dimensions (predictors)

| **Predictor** | **B** | **SE** | ***β*** | ***t*** | ***p*** | **95% CI** |
| --- | --- | --- | --- | --- | --- | --- |
| Extraversion | **-0.049** | **0.024** | **-0.187** | **-2.08** | **.04** | **(-0.095, -0.002)** |
| Agreeableness | -0.014 | 0.03 | -0.04 | -0.48 | .63 | (-0.073, 0.045) |
| Conscientiousness | -0.002 | 0.023 | -0.009 | -0.11 | .91 | (-0.048, 0.043) |
| Negative emotionality | -0.016 | 0.023 | -0.063 | -0.70 | .49 | (-0.061, 0.029) |
| Open mindedness | 0.057 | 0.03 | 0.17 | 1.89 | .06 | (-0.003, 0.117) |
| Model fit | *F*(5,136) = 1.49, *p* = .20, *R^2^* = .05, Adjusted *R^2^* = .02 | | | | | |

*Note:* B = unstandardised regression coefficient, SE = Standard error of B, *β* = standardised regression coefficient, CI = confidence intervals.

**Supplementary Table 5.** Multiple regression coefficients and model fit for predicting familiarity performance for expected events from the big five personality dimensions (predictors)

| **Predictor** | **B** | **SE** | ***β*** | ***t*** | ***P*** | **95% CI** |
| --- | --- | --- | --- | --- | --- | --- |
| Extraversion | 0.005 | 0.026 | 0.016 | 0.18 | .86 | (-0.046, 0.055) |
| Agreeableness | 0.010 | 0.032 | 0.026 | 0.30 | .76 | (-0.054, 0.074) |
| Conscientiousness | 0.034 | 0.025 | 0.116 | 1.35 | .18 | (-0.016, 0.084) |
| Negative emotionality | 0.031 | 0.025 | 0.112 | 1.23 | .22 | (-0.019, 0.08) |
| Open mindedness | 0.051 | 0.033 | 0.140 | 1.54 | .12 | (-0.014, 0.116) |
| Model fit | *F*(5,136) = 1.05, *p* = .39, *R^2^* = .04, Adjusted *R^2^* = .002 | | | | | |

*Note:* B = unstandardised regression coefficient, SE = Standard error of B, *β* = standardised regression coefficient, CI = confidence intervals.

**Supplementary Table 6.** Multiple regression coefficients and model fit for predicting recollection performance for expected events from the big five personality dimensions (predictors)

| **Predictor** | **B** | **SE** | ***β*** | ***t*** | ***P*** | **95% CI** |
| --- | --- | --- | --- | --- | --- | --- |
| Extraversion | **-0.060** | **0.027** | **-0.195** | **-2.19** | **.03** | (-0.114, -0.006) |
| Agreeableness | -0.022 | 0.035 | -0.052 | -0.62 | .53 | (-0.09, 0.047) |
| Conscientiousness | -0.031 | 0.027 | -0.098 | -1.16 | .25 | (-0.085, 0.022) |
| Negative emotionality | -0.049 | 0.027 | -0.163 | -1.83 | .07 | (-0.102, 0.004) |
| Open mindedness | 0.003 | 0.035 | 0.007 | 0.07 | .94 | (-0.067, 0.072) |
| Model fit | *F*(5,136) = 1.82, *p* = .11, *R^2^* = .06, Adjusted *R^2^* = .03 | | | | | |

*Note:* B = unstandardised regression coefficient, SE = Standard error of B, *β* = standardised regression coefficient, CI = confidence intervals.

**Supplementary Table 7.** Multiple regression coefficients and model fit for predicting familiarity performance for unexpected events from the big five personality dimensions (predictors)

| **Predictor** | **B** | **SE** | ***β*** | ***t*** | ***P*** | **95% CI** |
| --- | --- | --- | --- | --- | --- | --- |
| Extraversion | -0.026 | 0.024 | -0.093 | -1.06 | .29 | (-0.073, 0.022) |
| Agreeableness | 0.039 | 0.031 | 0.106 | 1.29 | .20 | (-0.021, 0.100) |
| Conscientiousness | 0.004 | 0.024 | 0.014 | 0.17 | .87 | (-0.043, 0.051) |
| Negative emotionality | **0.057** | **0.024** | **0.214** | **2.43** | **.02** | **(0.011, 0.104)** |
| Open mindedness | **0.078** | **0.031** | **0.222** | **2.52** | **.01** | **(0.017, 0.14)** |
| Model fit | *F*(5,136) = 2.72, *p* = .02, *R^2^* = .09, Adjusted *R^2^* = .06 | | | | | |

*Note:* B = unstandardised regression coefficient, SE = Standard error of B, *β* = standardised regression coefficient, CI = confidence intervals.

**Supplementary Table 8.** Multiple regression coefficients and model fit for predicting recollection performance for unexpected events from the big five personality dimensions (predictors)

| **Predictor** | **B** | **SE** | ***β*** | ***t*** | ***P*** | **95% CI** |
| --- | --- | --- | --- | --- | --- | --- |
| Extraversion | -0.019 | 0.025 | -0.067 | -0.76 | .45 | (-0.068, 0.03) |
| Agreeableness | -0.055 | 0.031 | -0.146 | -1.76 | .08 | (-0.118, 0.007) |
| Conscientiousness | -0.012 | 0.024 | -0.04 | -0.47 | .64 | (-0.06, 0.037) |
| Negative emotionality | **-0.071** | **0.024** | **-0.26** | **-2.93** | **.004** | **(-0.119, -0.023)** |
| Open mindedness | -0.016 | 0.032 | -0.045 | -0.51 | .61 | (-0.08, 0.047) |
| Model fit | *F*(5,136) = 2.38, *p* = .04, *R^2^* = .08, Adjusted *R^2^* = .05 | | | | | |

*Note:* B = unstandardised regression coefficient, SE = Standard error of B, *β* = standardised regression coefficient, CI = confidence intervals.

1. **Supplementary Figures**


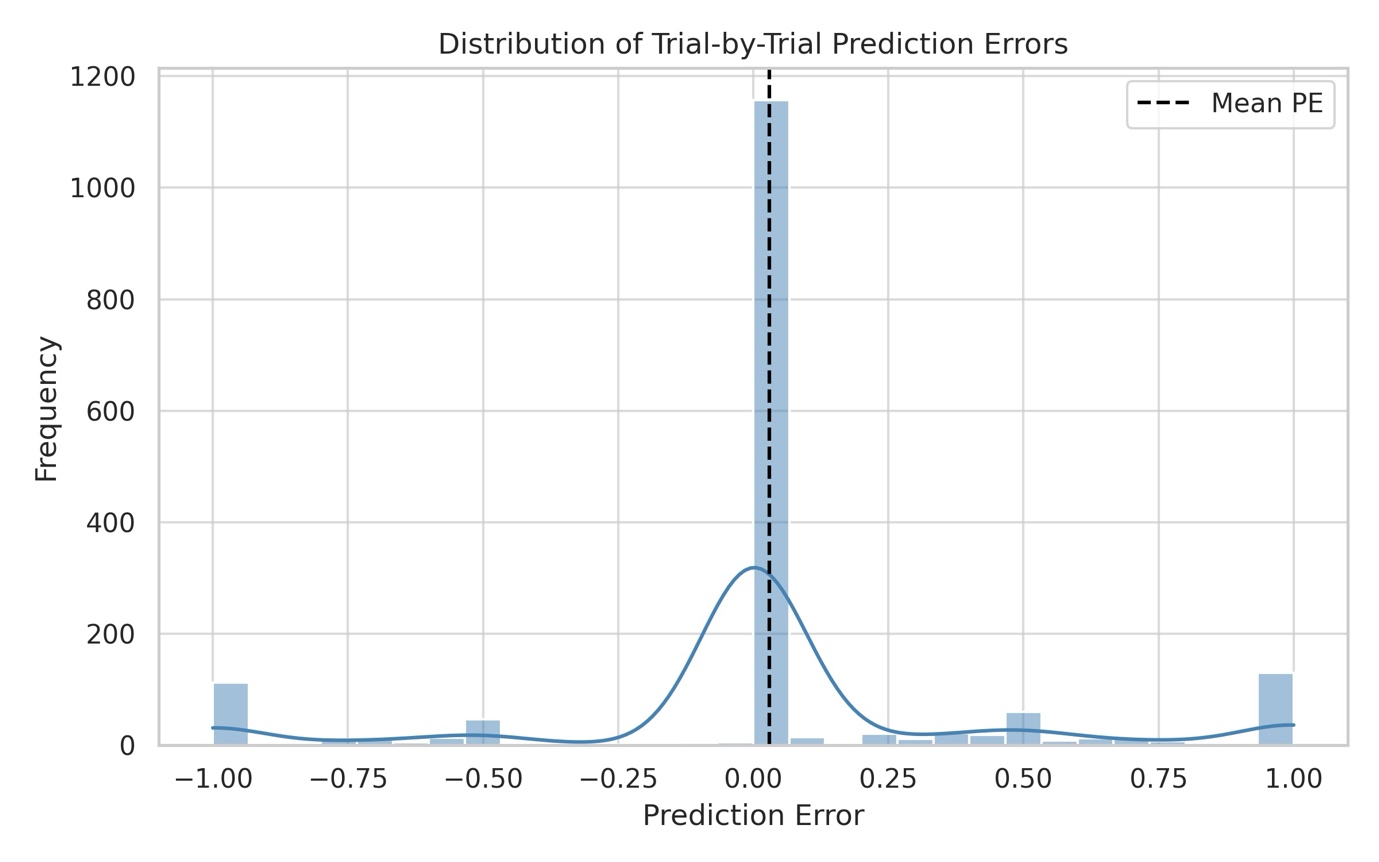
**Supplementary Figure 1.** Distribution of trial-by-trial prediction errors derived from the Rescorla–Wagner model. This histogram displays the distribution of prediction error (PE) values across all first-presentation learning trials in Experiment 1. PE was computed on each trial as the difference between the observed outcome (1 for correct, 0 for incorrect) and the model’s current expectation (associative strength, V) for the symbol–category pairing. The resulting distribution is broad and symmetrical, centred near zero, with no extreme outliers. This confirms that the model captured a wide range of expectancy violations, from large mismatches early in learning to minimal surprise once associations were established. The presence of values near zero reflects trials where participants’ expectations were well aligned with outcomes, indicating stable learning. These features validate the use of PE as a continuous, dynamic learning signal in subsequent analyses of memory performance and individual differences.

*Note:* Prediction error values of zero occur naturally in the Rescorla–Wagner model when a participant’s internal expectation fully aligns with the observed outcome, typically following repeated correct learning. These trials reflect successful learning and a lack of surprise, rather than an absence of meaningful updates. Their presence demonstrates that the model is functioning as expected, with associative strengths stabilising in response to consistent feedback. This is particularly likely given that learning rate α was fixed at 1.0, allowing rapid convergence.

**
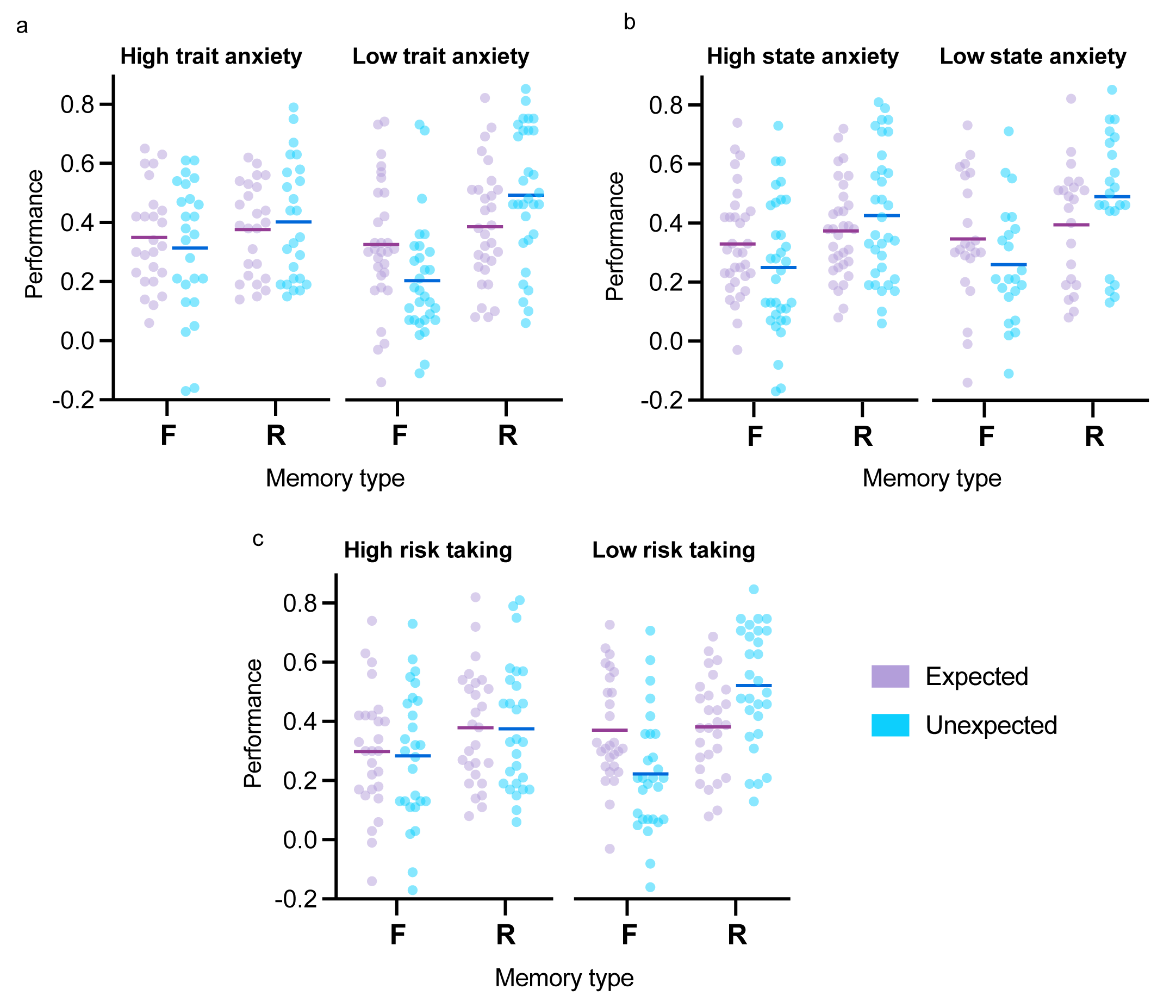
Supplementary Figure 2.** Scatterplots showing individual participant memory performance data for expected and unexpected stimuli, for participants with high and low trait anxiety (a), high and low state anxiety (b), and high and low risk taking (c). Each point represents a single participant; horizontal lines indicate condition means (± SEM). These plots correspond to the group effects illustrated in Figure 4.

**
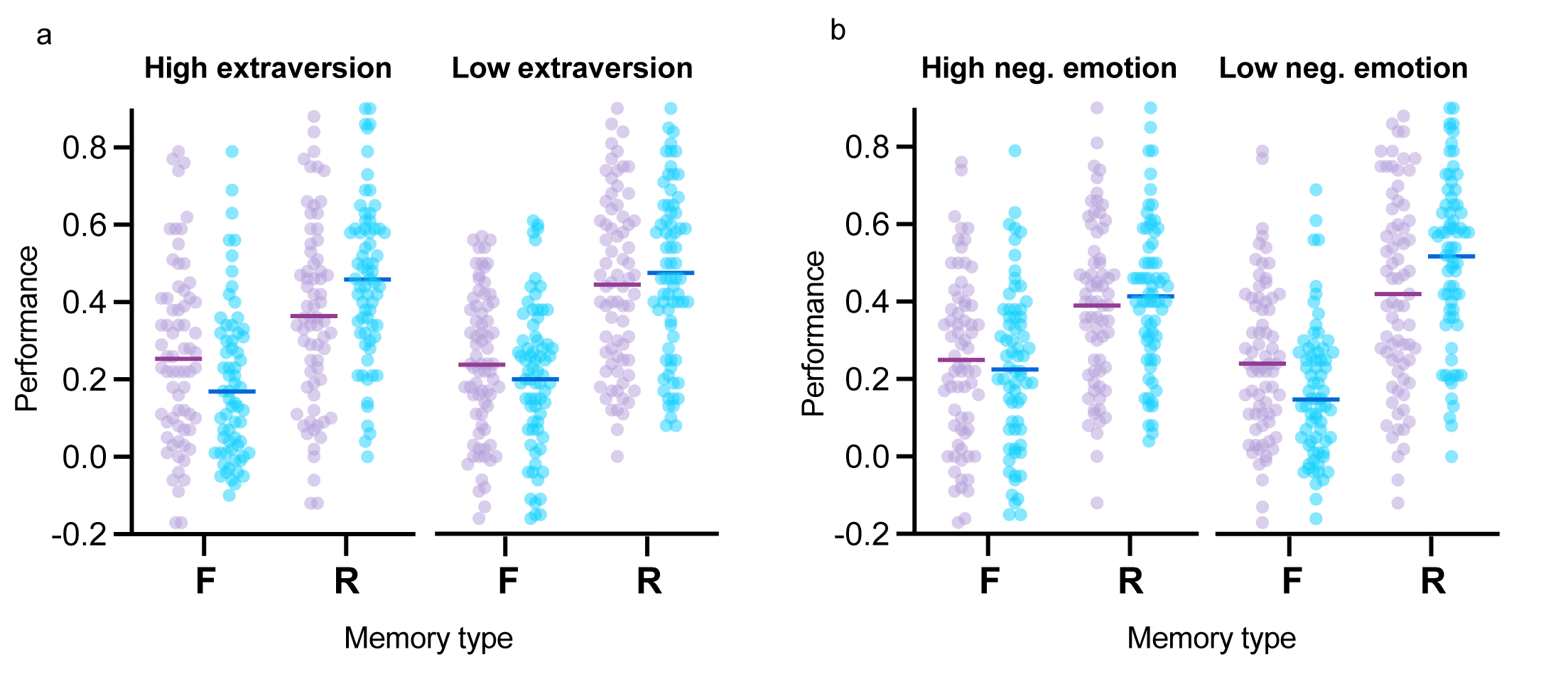
Supplementary Figure 3.** Scatterplots showing individual participant memory performance data for expected and unexpected stimuli, for participants with high and low extraversion (a) and high and low negative emotionality (b). Each point represents a single participant; horizontal lines indicate condition means (± SEM). These plots correspond to the group effects illustrated in Figure 6.

**
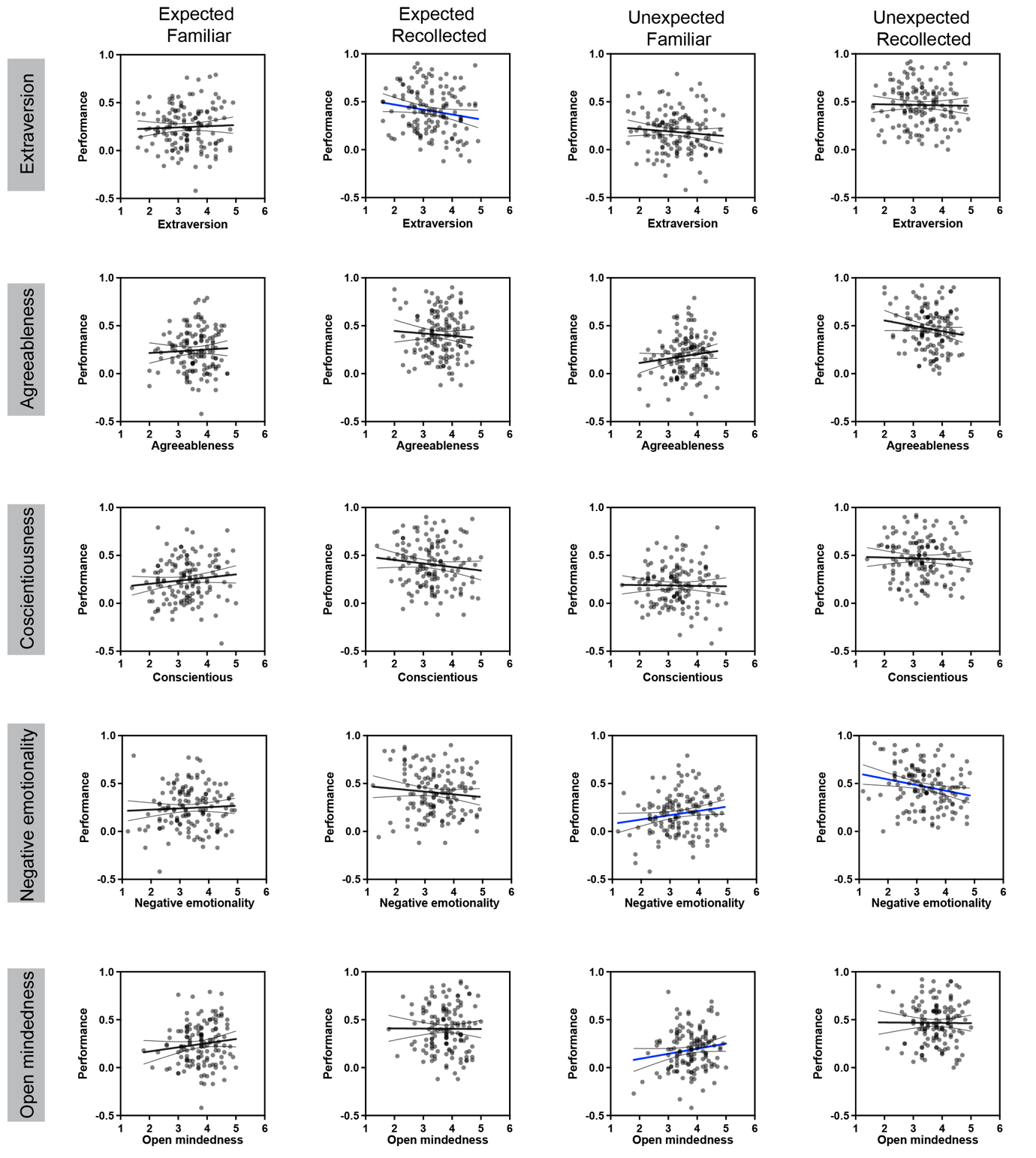
**

**Supplementary Figure 4.** Scatterplots with regression lines showing the relationship between memory performance and personality traits*.* Black regression lines indicate non-significant effects; blue lines indicate significant effects. Performance is calculated as hits minus false alarm rates, separately for familiarity and recollection responses across expected and unexpected stimuli.
